# Membrane protein megahertz crystallography at the European XFEL

**DOI:** 10.1038/s41467-019-12955-3

**Published:** 2019-11-04

**Authors:** Chris Gisriel, Jesse Coe, Romain Letrun, Oleksandr M. Yefanov, Cesar Luna-Chavez, Natasha E. Stander, Stella Lisova, Valerio Mariani, Manuela Kuhn, Steve Aplin, Thomas D. Grant, Katerina Dörner, Tokushi Sato, Austin Echelmeier, Jorvani Cruz Villarreal, Mark S. Hunter, Max O. Wiedorn, Juraj Knoska, Victoria Mazalova, Shatabdi Roy-Chowdhury, Jay-How Yang, Alex Jones, Richard Bean, Johan Bielecki, Yoonhee Kim, Grant Mills, Britta Weinhausen, Jose D. Meza, Nasser Al-Qudami, Saša Bajt, Gerrit Brehm, Sabine Botha, Djelloul Boukhelef, Sandor Brockhauser, Barry D. Bruce, Matthew A. Coleman, Cyril Danilevski, Erin Discianno, Zachary Dobson, Hans Fangohr, Jose M. Martin-Garcia, Yaroslav Gevorkov, Steffen Hauf, Ahmad Hosseinizadeh, Friederike Januschek, Gihan K. Ketawala, Christopher Kupitz, Luis Maia, Maurizio Manetti, Marc Messerschmidt, Thomas Michelat, Jyotirmoy Mondal, Abbas Ourmazd, Gianpietro Previtali, Iosifina Sarrou, Silvan Schön, Peter Schwander, Megan L. Shelby, Alessandro Silenzi, Jolanta Sztuk-Dambietz, Janusz Szuba, Monica Turcato, Thomas A. White, Krzysztof Wrona, Chen Xu, Mohamed H. Abdellatif, James D. Zook, John C. H. Spence, Henry N. Chapman, Anton Barty, Richard A. Kirian, Matthias Frank, Alexandra Ros, Marius Schmidt, Raimund Fromme, Adrian P. Mancuso, Petra Fromme, Nadia A. Zatsepin

**Affiliations:** 1 Biodesign Center for Applied Structural Discovery, Arizona State University, Tempe, AZ 85287-5001 USA; 2 School of Molecular Sciences, Arizona State University, Tempe, AZ 85287-1604 USA; 3 European XFEL GmbH, Holzkoppel 4, 22869 Schenefeld, Germany; 4 Center for Free-Electron Laser Science, Deutsches Elektronen-Synchrotron, Notkestrasse 85, 22607 Hamburg, Germany; 5 Department of Physics, Arizona State University, Tempe, AZ 85287-1504 USA; 6 Hauptman-Woodward Institute, 700 Ellicott St, Buffalo, NY 14203-1102 USA; 7 Department of Structural Biology, Jacobs School of Medicine and Biomedical Sciences, SUNY University at Buffalo, 700 Ellicott St, Buffalo, NY 14203-1102 USA; 8 Linac Coherent Light Source, SLAC National Accelerator Laboratory, Menlo Park, 94025 CA USA; 9 Department of Physics, Universität Hamburg, Luruper Chaussee 149, 22761 Hamburg, Germany; 10 The Hamburg Centre for Ultrafast Imaging, Universität Hamburg, Luruper Chaussee 149, 22761 Hamburg, Germany; 11 Deutsches Elektronen-Synchrotron, Notkestrasse 85, 22607 Hamburg, Germany; 12 Institute for X-Ray Physics, University of Göttingen, 37077 Göttingen, Germany; 13 Center Nanoscale Microscopy and Molecular Physiology of the Brain, Göttingen, Germany; 14 Biological Research Centre, Hungarian Academy of Sciences, Temesvári krt. 62, Szeged, 6726 Hungary; 15 Department of Biochemistry & Cellular and Molecular Biology, University of Tennessee at Knoxville, Knoxville, TN USA 37996; 16 Program in Energy Science and Engineering, University of Tennessee at Knoxville, Knoxville, TN USA 37996; 17 Department of Microbiology, University of Tennessee at Knoxville, Knoxville, TN USA 37996; 18 Lawrence Livermore National Laboratory, 7000 East Avenue, Livermore, CA 94550 USA; 19 University of Southampton, University Rd, Southampton, SO17 1BJ UK; 20 Hamburg University of Technology, Vision Systems E-2, Harburger Schloßstraße 20, 21079 Hamburg, Germany; 21 Department of Physics, University of Wisconsin-Milwaukee, 3135 N. Maryland Ave, Milwaukee, WI 53211 USA; 22 Department of Chemistry and Physics, La Trobe Institute for Molecular Science, La Trobe University, Melbourne, 3086 Victoria Australia; 23 Present Address: Department of Chemistry, Yale University, New Haven, CT 06520 USA; 24 Present Address: Deutsches Elektronen-Synchrotron, Notkestrasse 85, 22607 Hamburg, Germany; 25 Present Address: ARC Centre of Excellence in Advanced Molecular Imaging, Department of Chemistry and Physics, La Trobe Institute for Molecular Science, La Trobe University, Melbourne, 3086 Victoria Australia

**Keywords:** X-ray crystallography, X-ray crystallography, Nanocrystallography, Nanocrystallography, Structural biology

## Abstract

The world’s first superconducting megahertz repetition rate hard X-ray free-electron laser (XFEL), the European XFEL, began operation in 2017, featuring a unique pulse train structure with 886 ns between pulses. With its rapid pulse rate, the European XFEL may alleviate some of the increasing demand for XFEL beamtime, particularly for membrane protein serial femtosecond crystallography (SFX), leveraging orders-of-magnitude faster data collection. Here, we report the first membrane protein megahertz SFX experiment, where we determined a 2.9 Å-resolution SFX structure of the large membrane protein complex, Photosystem I, a > 1 MDa complex containing 36 protein subunits and 381 cofactors. We address challenges to megahertz SFX for membrane protein complexes, including growth of large quantities of crystals and the large molecular and unit cell size that influence data collection and analysis. The results imply that megahertz crystallography could have an important impact on structure determination of large protein complexes with XFELs.

## Abstract (web summary)

The European X-ray free-electron laser (EuXFEL) in Hamburg is the first XFEL with a megahertz repetition rate. Here the authors present the 2.9 Å structure of the large membrane protein complex Photosystem I from *T. elongatus* that was determined at the EuXFEL.

## Introduction

The advent of hard X-ray free-electron lasers (XFELs) and emergence of serial femtosecond crystallography (SFX)^1,2^ has enhanced X-ray protein crystallography. In SFX, the extreme XFEL brilliance and ultrashort (fs scale) pulse duration enable atomic-resolution structure determination of proteins at room temperature (RT) from micron-sized protein crystals, while outrunning structure-altering (secondary) radiation damage.

Since the initial proof-of-principle serial crystallography experiments^1,2^ performed in 2009 at the first hard XFEL, the Linac Coherent Light Source (LCLS) at SLAC National Accelerator Laboratory, XFEL technologies have been further developed that include novel techniques to improve the growth of nano-/microcrystals, deliver samples, and analyze SFX data. The implementation of time-resolved SFX^3–9^ garnered interest from the structural biology community and increased the demand for XFEL beamtime. To make XFEL technology available for these experiments, strategies to increase the data collection speed are needed. A prime approach to achieving this is the use of high X-ray pulse repetition rates, which have been realized at the European XFEL (EuXFEL).

Since the opening of LCLS, four more XFELs have begun user operation: SPring-8 Angstrom Compact Free-Electron Laser (SACLA) in Japan opened in 2011, Pohang Accelerator Laboratory X-ray Free-Electron Laser (PAL-XFEL) in South Korea opened in 2016, the EuXFEL in Germany opened in 2017, and SwissFEL in Switzerland began user operation recently. At PAL-XFEL, SACLA, SwissFEL, and LCLS, the data acquisition rates, limited by the XFEL pulse rate, are 30–120 Hz (Supplementary Table 1).

Powered by a superconducting linear accelerator, the EuXFEL is the first MHz repetition rate hard XFEL, with a bunch structure designed to deliver up to 27,000 pulses per second. Typically, XFELs produce uniformly spaced X-ray pulses at a constant repetition rate. However, the EuXFEL delivers XFEL pulses in 10-Hz pulse trains with up to 2700 pulses per train planned. User-assisted commissioning started in September 2017. In its first run, the EuXFEL delivered pulse trains at 10 Hz where each train contained up to 60 pulses, separated by 886 ns (~1.128 MHz). In run 2, the rate was increased to 120 pulses per train, thus delivering 1200 pulses per second, and correspondingly, at least a tenfold increase in the data collection rate compared with other hard XFELs. The term MHz crystallography was first introduced in the reporting of SFX at the EuXFEL with concanavalin A, concanavalin B, and lysozyme by Grünbein et al.^10^ and on lysozyme and β-lactamase by Wiedorn et al.^11^, where MHz refers to the repetition rate within a pulse train.

MHz pulse rates bring new challenges. In SFX, the sample is delivered in a serial way to the beam, commonly in a jet of crystals in their mother liquor^2^. It must be rapidly replenished between the XFEL pulses, removing the sample destroyed by the previous pulse. Time separation between pulses within a train at the EuXFEL is so brief that the sample must be replenished at least 9000 times faster than required at the LCLS where pulses are separated by 8.3 ms. The first two publications reporting SFX experiments at the EuXFEL demonstrated that the sample can be effectively replenished in 886 ns between the X-ray pulses^10,11^ with a 15 -µm diameter at full-width half-maximum (FWHM) beam focus by using a gas-focused micron-scale liquid stream (with a gas-dynamic virtual nozzle, or GDVN)^12^ with a velocity of at least 50 m/s^10,13^, and that molecular structures can be determined from data collected at the EuXFEL^10,11^.

However, current sample injection technology results in substantial sample wastage between pulse trains^11^. This poses a challenge for diminishing sample consumption, despite the MHz repetition rate within a pulse train. The volume of data collected per volume of sample consumed will, however, increase significantly with the anticipated peak performance of the EuXFEL of up to 2700 pulses per train, and the upcoming LCLS-II, which will provide up to 1 million pulses per second^14^. Note that the repetition rate of the XFEL is not the only limitation for the rate of data collection at MHz XFELs. Currently, the fastest X-ray detector is the newly developed Adaptive Gain Integrating Pixel Detector (AGIPD), which can collect up to 352 images per train (potentially storing 3520 images per second)^15,16^.

The required fast jet speed and high sample consumption was a challenge for our SFX experiment on the large membrane protein complex PSI at the EuXFEL. Previous MHz SFX experiments were performed with commercially available proteins (i.e., lysozyme and jack bean meal powder)^10^ or proteins that could be heterologously overexpressed in *Escherichia coli* in large amounts (e.g., BlaC)^11^. However, it is difficult to express and purify membrane proteins in sufficient quality, quantity, and stability for X-ray crystallography, accounting for a major bottleneck in structural biology. To date, fewer than 1000 unique membrane protein structures have been solved (see http://blanco.biomol.uci.edu/mpstruc/ for a current update on membrane protein structures). The problem is more pronounced for multi-subunit and ligand-rich membrane protein complexes, as they require specific cell machinery for membrane insertion and complex assembly. In the case of PSI, a complicated system of proteins is required for synthesis of cofactors and assembly of the complex^17^. This is why most structures from large membrane protein complexes, including PSI, can only be determined from native protein complexes, isolated from their natural host cells.

Here, we report the megahertz (MHz) SFX study of a large membrane protein complex at the EuXFEL, and discuss challenges that accompany the MHz repetition rates and how they were resolved. We prepared large batches of membrane proteins in sufficient amounts for the XFEL study and grew billions of uniform cyanobacterial Photosystem I (PSI) microcrystals that were of similar size to the XFEL beam focus, by crystallization at low ionic strength, a method that allowed the facilitation of fast sample delivery required for high X-ray pulse repetition rates. In addition, we describe how we overcame the challenges in the data collection and analysis of diffraction data from protein crystals with large unit cells that allowed us to determine the room- temperature (RT) structure of PSI at 2.9-Å resolution.

## Results

### Photosystem I isolation and crystallization

**Fig. 1:**
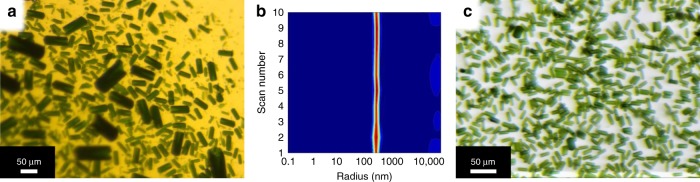
Crystals of PSI. **a** Variable-size PSI crystal distribution, grown by ultrafiltration. **b** DLS (ten 18-s scans, numbered consecutively from 1 to 10, “Scan Number”) of nanocrystals with uniform size distribution used for seeding. **c** PSI crystals of uniform size of 5 × 5 × 15 µm grown by using the RAMS method

Here we briefly summarize the procedures and challenges of membrane protein isolation and crystallization for MHz SFX experiments. PSI was isolated from the natural thermophilic cyanobacterium *Thermosynechococcus elongatus*. The cells were grown in a 120-L photobioreactor (Supplementary Fig. 1a), and PSI was isolated by a 36-h multistep isolation procedure, including membrane isolation, detergent solubilization, ion exchange chromatography (Supplementary Fig. 1b), and crystallization as a final purification step (for more details see the “Methods” section). In total, 1000 mg of PSI was isolated and crystallized for XFEL experiments. PSI cannot be frozen at any step during the isolation and crystallization process, so protein was freshly isolated before the beamtime and recrystallized on-site. The first crystallization step was performed by concentrating the sample using ultrafiltration at low ionic strength (additional experimental details can be found in the Methods section). This produced a crystal suspension with a broad size distribution from ~200 nm to 100 µm (Fig. 1a). The crystals could be sorted by size through sequential timed sedimentation, to separate the nanocrystals from larger crystals^18^. The nanocrystals were used as seeds for the final crystallization step. For the final crystallization, the larger crystals were dissolved by increasing the ionic strength, leading to a highly concentrated protein solution of 100 mg/mL PSI. Initial testing of optimal crystallization conditions was performed at low ionic strength (Supplementary Fig. 2).

To achieve uniform crystals for XFEL delivery, growth of crystals from the seeds must occur rapidly, thereby outrunning new nucleation events. This was achieved by a method that we term rotational agitated mixing with seeds (RAMS, see also Methods and Supplementary Fig. 3). Nanoseeds of uniform size (confirmed by dynamic light scattering (DLS), see Fig. 1b) were added to a precipitant solution consisting of the low ionic-strength buffer, before crystallization was induced by mixing of the seeded precipitant solution with small droplets of highly concentrated PSI (at 100 mg/mL). Incubation for 1 h at RT in the dark resulted in the growth of uniformly sized PSI crystals (15 × 5 × 5 µm).

### Sample delivery

PSI microcrystals were delivered to the EuXFEL beam in a liquid jet produced by a GDVN^12^ at a jet speed of 50 m/s, which is required to replenish the sample in 886 ns between the X-ray pulses of the EuXFEL^10,11,13^. A high flow speed was achieved with a flow rate of 20 µL/min in glass GDVNs with an inner diameter (ID) of 50 µm. We accomplished successful sample delivery and replenishment by using 50-µm-ID nozzles due to the low-viscosity low ionic-strength buffer, high-quality manually ground glass GDVNs, and the avoidance of connections that could restrict the flow diameter and cause clogging.

### Data collection and analysis

SFX data were collected at the SPB/SFX instrument of the EuXFEL^19,20^ by using X-rays of ~9.3 keV with an average pulse energy of 0.7–1 mJ, which corresponds to 4.7–6.7 × 10^11^ photons/pulse upstream of the SPB/SFX hutch. The flux at the sample position was estimated to be reduced by ~50%, and the focal spot diameter was 16 ± 4 µm^2^ at FWHM. The XFEL pulses were delivered in 30-pulse trains at 10 trains per second, with 886 ns between pulses within the trains (1.128 MHz), corresponding to an effective measurement rate of 300 pulses per second overall. The pulse duration was estimated from the electron bunch length to be ~50 fs. This study focuses on the dark structure of PSI, which corresponded to the first ten images from each pulse train.

The data were collected with the AGIPD at three different distances (16.8, 23.3, and 32.7 cm from the sample) to assess indexing rate, quality (by providing sufficient peak separation), and resolution. Most data were collected at a detector distance of 23.3 cm with a resolution of 2.6 and 3.0 Å at the AGIPD edges vertically and horizontally, respectively. At 23.3 -cm distance, the minimum peak separation was ~5 pixels (4 pixels for 16.8 cm).

**Fig. 2:**
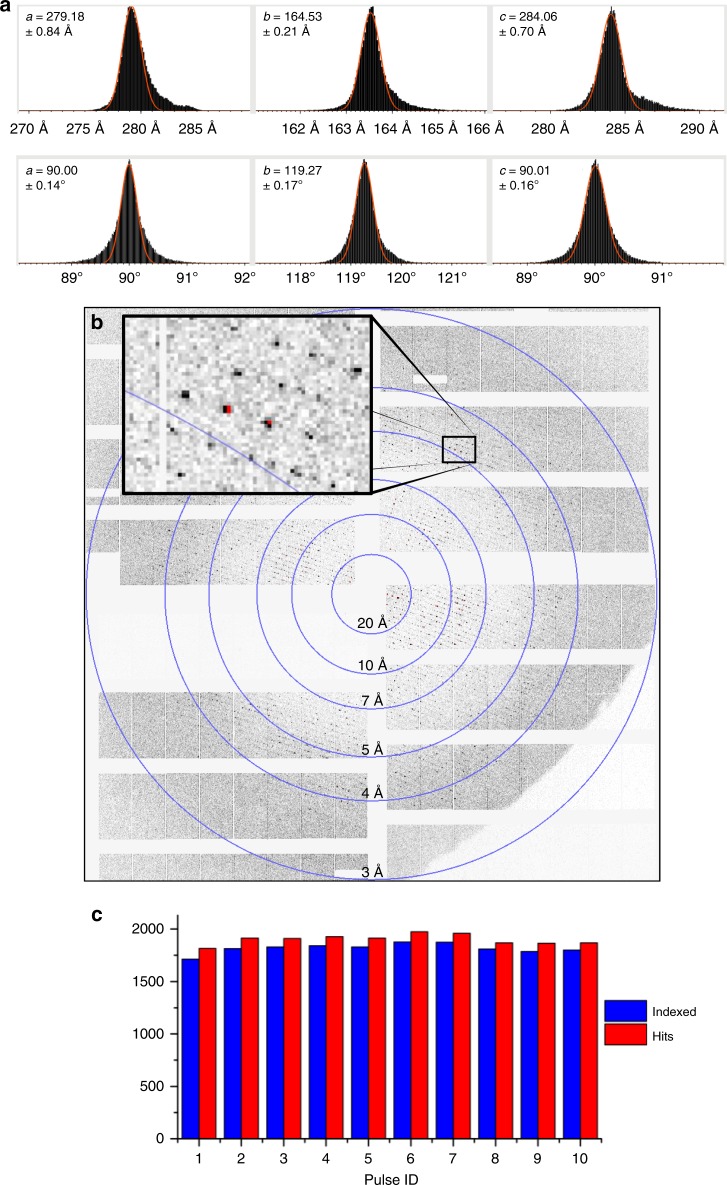
Unit cell distribution, diffraction pattern, and indexing rates from PSI MHz SFX. **a** Unit cell distributions of PSI microcrystals showing narrowly distributed monoclinic unit cells. The red line shows a Gaussian function fit to the unit cell constant distribution, and the corresponding peak value is listed in each subpanel. **b** Representative X-ray diffraction pattern with pixels in high-gain mode shown in black, and medium- or low-gain mode shown in red. Resolution rings are shown and labeled accordingly. **c** The number of hits (red) and indexed patterns (blue, ~93% of hits) for each pulse

The data acquisition was monitored^21^ in real time by using the online interface OnDA^22^. The data set reported here consists of 7,719,186 diffraction patterns from which 76,850 were identified as crystal diffraction patterns (hits) by Cheetah^23^ corresponding to an average hit rate of ~1%. For the analysis, only runs with detector distances 23.3 and 32.7 cm were considered giving 59,012 patterns (hits, Table 1) in total. This hit rate is expected at the given crystal density of 1.68 × 10^8^ crystals/mL, which was experimentally determined by counting the crystals in a cell-counting chamber. This was the maximal PSI crystal density that could be achieved with this crystal size (5 × 5 × 15 µm); higher crystal densities led to clogging of the 50-µm-ID nozzles. With a jet speed of 50 m/s, a jet diameter of 5 µm, a beam diameter of 15 µm, and using conservative peak finder settings (i.e., only peaks consisting of at least 3 pixels), the maximal possible hit rate of 1.7% matched well to the observed hit rate of ~1%. A Gaussian unit cell distribution was observed (Fig. 2a) in the collected data. A typical diffraction pattern of PSI with close spot separation in the *a* and *c* directions is shown in Fig. 2b.

**Table 1:** Data collection statistics for PSI MHz SFX at the EuXFEL

Detector distance (cm)	32.7	23.3	Combined	Dark-state data (pulses 1–10 only)
Hit rate (%)	1.0	1.0	~1	~1
Hits	7900	51,112	59,012	19,023
Indexed patterns (30 pulses/train)	7403	47,377	54,780	18,176
Indexing rate	94%	93%	93%	96%
Resolution at the edge of the detector (Å), horizontal, vertical	4.1, 3.5	3, 2.6	Various	Various
Minimum peak separation (pixels)	7.6	5.4	Various	5–7

The data were used from two different detector positions. Only the first ten pulses of a given train contributed to the dark PSI structure determined here

The hit rate remained relatively constant throughout the pulse sequences (Fig. 2c), indicating that the sample had been fully replenished between the pulses. The successful collection of diffraction data from PSI microcrystals was facilitated by the AGIPD’s feature of three individual gain stages (for details see Henrich et al.^15^). The diffraction pattern shown in Fig. 2b highlights reflections and pixels in the high- (black) and medium- (red) gain settings.

We observed 19,023 hits from the first ten pulses of the pulse train, representing diffraction images of the dark state of PSI. From these images, 18,176 (93%) were indexed in CrystFEL^24,25^ by using the indexing program Xgandalf^26^.

**Fig. 3:**
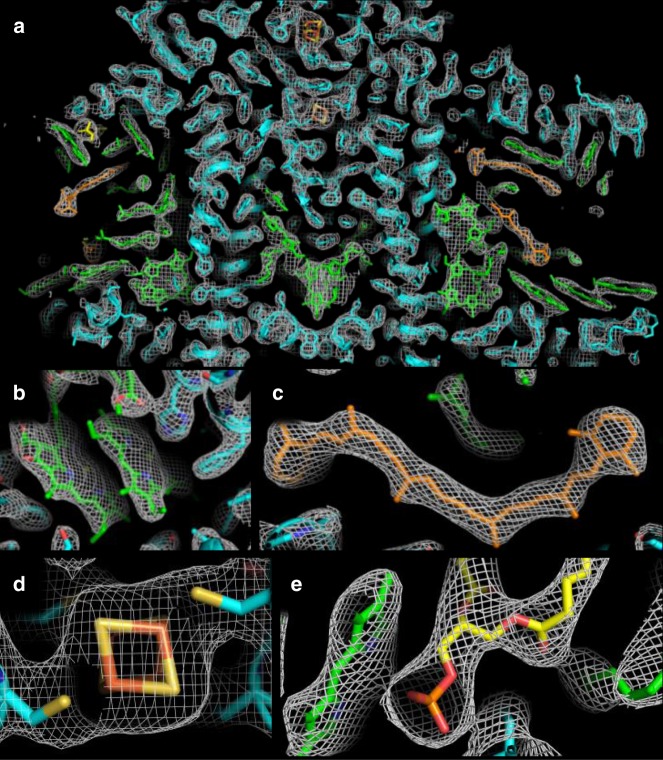
Electron density map (2Fo–Fc at 1.5σ) and model of various PSI structural elements of the XFEL structure of PSI. In all images, protein is colored cyan, chlorophyll (Chl) molecules are colored green, β-carotenes are colored orange, and lipids are colored yellow. In panels **b**–**e**, nitrogen atoms are colored blue, oxygen atoms are colored red, and magnesium atoms are colored bright green. **a** A slice through the center of electron density of a monomer of PSI is shown, **b** the electron density of the “special pair” of Chls, P_700_, **c** a β-carotene molecule, **d** the 4Fe–4S cluster, F_X_, and **e** the phosphatidylglycerol lipid headgroup axial coordination of a Chl molecule

Phases were obtained with CCP4’s implementation of Phaser^27^ by using the known X-ray structure of PSI from *T. elongatus* that was determined by using synchrotron radiation (PDB ID = 1 JB0^28^) as a starting model for molecular replacement. The final data collection and refinement statistics for the PSI structure are shown in Table 2, and more details on the data analysis statistics can be found in the Methods and Supplementary Figs. 4–6. The generated electron density map is of high quality with important cofactors being well-resolved (see Fig. 3). For comparison of the SFX data with standard synchrotron-collected data, we additionally collected a data set from a large single PSI crystal, cutting the data at 2.9 Å to be comparable to the XFEL data set’s resolution cutoff. The data statistics for both data sets are shown in Table 2, and more details on crystallization procedures, as well as data collection and analysis for the synchrotron-collected data are included in Supplementary Note 1. The processing of the cryogenic synchrotron structure also confirmed the P2_1_ space group determined from the RT XFEL data (discussed further below).

**Table 2:** Crystallography data and refinement statistics for the XFEL and synchrotron data

Data collection	XFEL	Synchrotron
Space group	P2_1_	P2_1_
Dimensions: a, b, c (Å)	279.2, 164.5, 284.1	278.5, 165.1, 283.4
Dimensions: α, β, γ (°)	90, 119.3, 90.0	90, 119.4, 90
Number of hits	19,023	180
Indexed patterns	18,176	180
Resolution (Å)	64.32–2.9 (3.0–2.9)	48.9–2.9 (3.0–2.9)
I/σI	3.61 (0.52)	7.1 (1.9)
Completeness (%)	100 (100)	99.6 (98.3)
CC*	0.97 (0.31)	0.991 (0.890)
CC_1/2_	0.88 (0.051)	0.633 (0.655)
Multiplicity	213 (122)	3.6 (1.9)
R_split_ (%)	22.07 (241.88)	N/A
R_merge_	N/A	0.136 (0.328)
Wilson B-factor (Å^2^)	51.3	55.38
Total number of reflections	104,759,101 (5,776,349)	1,769,616 (91,759)
Number of unique reflections	492,851 (47,459)	492,002 (47,514)
*Refinement*		
R_work_/R_free_	0.30/0.33	0.30/0.34
Number of atoms	72,533	72,738
Mean B-factor for all atoms (Å^2^)	60	47
Mean B-factor for protein (Å^2^)	61	46
Mean B-factor for ligands (Å^2^)	60	47
Mean B-factor for solvent (Å^2^)	33	32
CC_work_	0.790 (0.161)	0.759 (0.606)
CC_free_	0.790 (0.186)	0.697 (0.496)
*RMSD*		
Bond lengths (Å)	0.02	0.02
Bond angles (°)	2.38	2.44
Ramachandran allowed (%)	98.16	98.19

Where two values are quoted, these are the average overall resolution of all shells and the highest resolution shell (in parentheses)

The RT XFEL model and the cryogenic synchrotron model were found to be similar at this resolution (see superposition RMSD values in Supplementary Table 2). Long-range measurements on the structure showed that the RT XFEL structure is slightly expanded relative to the cryogenic synchrotron structure (Supplementary Fig. 7). Unsurprisingly, the RT XFEL structure has higher B-factors than the cryogenic synchrotron structure (Supplementary Fig. 8).

## Discussion

**Fig. 4:**
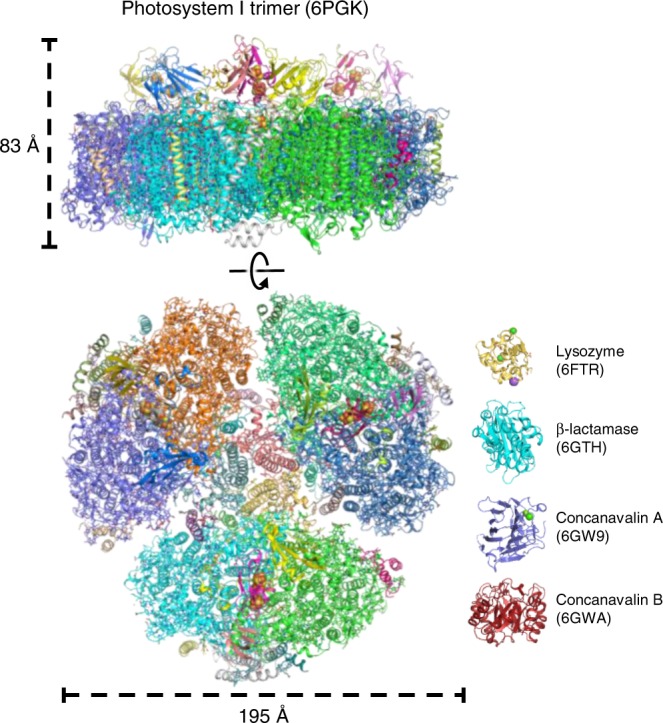
Comparison of SFX structures from the EuXFEL. The structure of the trimeric PSI determined in this study is shown to scale with the four protein structures previously solved at the EuXFEL by using MHz repetition rates for comparison. Views from the membrane plane (top) and membrane normal (bottom) are shown for the PSI trimer, with major and minor axes denoted. Protein subunits are colored individually

The first structure determined at the EuXFEL was of small single-domain soluble proteins with comparatively small unit cells^10,11^. Figure 4 shows the comparison of the trimeric PSI structure determined in this study with the structures of the four small proteins whose structures were previously solved via MHz crystallography at the EuXFEL^10,11^. In addition, we provide a table comparing molecule size, crystal size, and the number of unit cells/crystal in Supplementary Table 3. While the structure determination of these proteins with MHz repetition rates was challenging, determining the structure of the PSI trimer, a very large membrane protein complex posed further challenges ranging from isolation and crystallization to data collection, processing, and structure refinement, which are discussed here.

PSI crystallization was performed by decreasing the ionic strength. Low ionic-strength crystallization by dialysis with micro- and macroseeding was established for the growth of large single crystals of PSI that formed the basis of the previously solved 2.5-Å synchrotron structure of PSI^28,29^. This strategy is seldomly used in macrocrystallography as low ionic phase space is difficult to obtain by vapor diffusion, where all buffers and salts in the protein solution are concentrated. Low ionic-strength crystallization has several benefits for SFX: (i) crystallization at low ionic strength avoids harsh conditions or precipitants. (ii) It is fully reversible, so crystals can be dissolved and recrystallized multiple times to reach the desired crystal size distribution. (iii) These conditions result in a low-viscosity solution, close to that of water, allowing for high jet speeds to be reached without high back pressure. (iv) In the absence of salt and high-viscosity precipitants like polyethylene glycol (PEG), crystal slurries tend to clog less. (v) It avoids a common problem of SFX data collection, in which the impact of the X-ray beam on the sample jet leads to the accumulation of buffer components like salt or PEG, forming stalactites on the nozzle tip and stalagmites in the catcher that grow into the interaction region; these formations endanger the detector due to strong diffraction from the PEG or salt crystals and disturb the GDVN gas and sample stream. (vi) It is ideal for time-resolved studies, as the absence of PEG and salt reduces the amount of debris on the windows of the sample catcher used for sample visualization and in-coupling of the optical laser. More insight regarding low ionic-strength crystallization can be found in Supplementary Note 2. It should be noted that the settling rate of PSI crystals in low ionic-strength buffer depends on the crystal size and density. Thus, we used an anti-settling device^30^ developed for the SPB/SFX instrument to prevent settling of the crystals in the sample reservoirs during data collection.

In SFX, crystals are ideally delivered to the interaction region at a rate equal to or greater than the X-ray pulse repetition rate whether for liquid jets, fixed targets, or other sample delivery methods^31^. The first published results from EuXFEL experiments show that a jet speed of at least 50 m/s is required to replenish the sample volume between X-ray pulses separated by ~886 ns, which can currently only be reached by injection by using the GDVN^32^ or the double-flow focused nozzle systems^33^. Low sample consumption injection systems developed for membrane protein microcrystal delivery, like the high-viscosity injector^34^, are currently too slow to be used at the MHz repetition rate produced at the EuXFEL. The high speed and sample flow rates required for sample injection present a challenge for proteins that are difficult to express and isolate in large amounts, with a large fraction of sample going to waste in between pulse trains. Only with exceptionally large efforts and large-volume bioreactor culture capability were we able to isolate 1000 mg of PSI from the native sources for this experiment. Ongoing research into pulse injectors^35,36^ and future developments in accelerator, detector, and computer technology may potentially alleviate this requirement in the future.

The adaptive gain switching of individual pixels provides the AGIPD with a large dynamic range, which is critical for accurate intensity measurement in crystallography, and was important for the data collection of PSI crystals that have a large unit cell. With the AGIPD’s multiple gain stages, data can be collected at full flux of the XFEL, where weaker high-resolution reflections are detected in high-gain mode, increasing their contribution to the high-resolution shell completeness. In contrast, intense low-resolution reflections are collected in medium- (or low-) gain mode, allowing for accurate determination of their intensity without the problem of saturation, leading to improved accuracy in the low-resolution shell. Figure 2b shows the distribution of Bragg peaks with those in high-gain mode in white and medium- or low-gain mode indicated in red. Supplementary Fig. 4 shows the high dynamic range of individual pixels in integrated Bragg spots from all indexed patterns. The limited size, however, of the currently installed AGIPD makes accurate spot location and indexing for samples with very large cells difficult and may preclude collection of high-resolution data. A 4-megapixel AGIPD is currently in the production stage for crystallography applications^16^.

Indexing crystallography data from crystals with large unit cells is not trivial. Multi-panel detector geometry optimization to subpixel accuracy becomes more critical than for samples with small unit cells because the distance between the Bragg peaks is only a few pixels (when the detector is sufficiently close to record high resolution). We used the program geoptimiser^37^ to optimize the detector geometry including 16 individual distances to each 128 × 512-pixel panel. Accurate peak selection and integration requires well-separated, sharp peaks. CrystFEL’s *indexamajig*^24^ integrates peaks in 2D on each diffraction pattern. We used the three-ring integration method in which the outer annulus around each peak is used to calculate the local background and sigma. Integration radii were 2, 3, and 6 pixels to account for the small spot spacing.

Previous synchrotron-based structures of PSI from *T. elongatus*^28,38–40^ and the first SFX structure of PSI at 8-Å resolution^2^ were determined in space group P6_3_ with only one monomer of the trimeric PSI in the asymmetric unit (where the threefold trimer axis was a crystallographic axis). The unit cell dimensions were reported to be a = 286 Å, b = 286 Å, c = 167 Å, α = 90°, β = 90°, and γ = 120° from a 4-Å resolution data set collected at RT at a synchrotron^41^, and a = 281.0 Å, b = 281.0 Å, c = 165.2 Å, α = 90°, β = 90°, and γ = 120° from a 2.5-Å resolution data set collected under cryogenic conditions^28^. We originally attempted to analyze the EuXFEL data set both in space group P6_3_ and without space group constraints. The data processing without space group constraints led to significantly improved statistics when data were merged in space group P2_1_. By processing the SFX EuXFEL data set in the space group P2_1_ we obtained unit cell constants of a = 279.2 Å, b = 164.5 Å, c = 284.1 Å, α = 90°, β = 119.3°, and γ = 90°.

We grew large PSI crystals by using the original crystallization and freezing procedures, and data sets were collected at the Advanced Photon Source (APS) under cryogenic conditions to 2.9-Å resolution (Table 2). The evaluation of this synchrotron data (Supplementary Text 1) confirmed the space group P2_1_. The comparison of the data statistics of the RT EuXFEL and cryogenic synchrotron data sets is shown in Table 2. The spatial arrangement of PSI molecules in space groups P6_3_ and P2_1_ is very similar; the membrane-integral section of the PSI trimer is not oriented exactly parallel to the plane of the two long unit cell axes in the crystals. As the trimeric symmetry of the molecule is noncrystallographic, the space group is (pseudohexagonal) monoclinic P2_1_. The packing of PSI in the space group P2_1_ is shown in Supplementary Fig. 9. The quality of the data obtained from the EuXFEL experiment described here, as well as improved data evaluation software, allowed us to detect the difference between the two space groups more precisely. In space group P2_1_, three times more indexed images are required than in space group P6_3_, and thus the number of unique reflections is increased by a factor of 3.

The only other published PSI structure solved by SFX was determined at 8-Å resolution in one of the first experiments at the LCLS^2^. Although this effort led to the emergence of SFX for structural biology, the limited resolution only allowed for the identification of secondary structure elements such as α-helices and the electron-dense 4Fe–4S metal clusters. At 2.9-Å resolution, most amino acid side chains, hydrocarbon substituents, and even some well-conserved water molecules have been assigned with confidence (Fig. 3).

In the previously solved structure of PSI from *T. elongatus*^28^, the three monomers that comprise the biologically relevant trimeric complex are perfectly equivalent, a result of the structure being solved in space group P6_3_ that imposes crystallographic symmetry on the individual monomers, with one monomer in the asymmetric unit. In the P2_1_ space group, the asymmetric unit contains the entire PSI trimer; this presented the opportunity to apply NCS^42^ within the refinement strategy, which has the potential to further improve the electron density map. However, when NCS was applied, the quality of the electron density map and its associated statistics did not improve. It was recently shown in the structure of PSI from another cyanobacterium, *Synechocystis* sp. PCC 6803, that processing the data without imposed symmetry in the space group P2_1_, although quite different from the crystal packing reported here, resulted in apparent differences between the monomers of the trimer, allowing the assignment of extra lipid molecules^43^. Supplementary Fig. 9 shows the crystal packing solved from the *T. elongatus* PSI XFEL data presented here, and Supplementary Fig. 10 shows the crystal packing reported from the *S*. sp. PCC 6803 structure. In the structures solved here, only minor differences were identified when comparing the electron density map and the resultant models for individual monomers (i.e., different rotamers for small side chains and slightly different Chl tail orientations, Supplementary Fig. 11), and no extra cofactors were identified (likely because the data are of lower resolution than the structure of PSI from *S*. sp. PCC 6803). However, long-range measurements (i.e., those spanning the entire complex) show that one of the three monomers of the trimer protrudes slightly away from the center relative to the other two monomers (Supplementary Fig. 7). Because the cryogenic synchrotron structure solved in P2_1_ has lower B-factors (Supplementary Fig. 8) and is slightly compacted relative to the RT XFEL structure (Supplementary Fig. 7), this protrusion is less obvious in the former that speaks to the importance of understanding the structures of proteins at biologically relevant temperatures over cryogenic structures, a characteristic of XFEL-derived crystallographic data. While it may be that this observed asymmetry is an artifact of crystallization, a biological origin is also conceivable. In addition to the aforementioned asymmetry observed in PSI from *S*. sp. PCC 6803, it has recently been shown that asymmetry is observed in the *T. elongatus* PSI trimer when ferredoxin is bound (ferredoxin is the natural electron acceptor from PSI). It could be that in the assembly mechanism, two PSI monomers associate first but cause steric hindrance for the final monomer’s insertion, disrupting the perfect C3 symmetry. We think it likely that processing higher-resolution data in the same space group will, in the future, enable the discovery of potentially important biological insights, especially with data collected from XFELs where the sample is not X-ray damaged.

Serial MHz crystallography holds potential for fast and accurate data collection at RT that is free of secondary X-ray damage. Its use, with concomitant improved methodology and tailored data analysis, may lead to the discovery of numerous structures of macromolecules in near-native states. With accelerated data collection and decreased sample consumption, it will soon be possible to obtain the high-multiplicity SFX data required for time-resolved studies at multiple time points in less time than is currently possible. The quality of merged SFX data is dependent on multiplicity, especially for larger, complex samples, for which collecting larger data sets is important. Larger data sets are also required to resolve small or subtle structural differences in time-resolved sequences.

The challenge of producing sufficient sample volumes for proteins that are difficult to express, isolate, and crystallize for MHz serial crystallography applications will be ameliorated when the EuXFEL and the future LCLS-II reach their full pulse-rate capabilities. The EuXFEL’s anticipated collection rate of 3520 frames per second with the AGIPD will enable the collection of complete SFX data sets within a few minutes as compared with hours currently. The prospect of continuous MHz pulse rates at future facilities such as the LCLS-II-HE offers even greater potential. Data acquisition and storage will then be the limitation of high-frequency XFELs, necessitating further detector development for online data processing and new avenues in high-throughput data transfer and storage to optimize their use^14^. With improved detector capabilities, further increase in repetition rates, and improved crystallization procedures, SFX can benefit from MHz crystallography at XFELs to determine molecular movies of various biologically relevant processes including ligand binding, substrate screening, and light-induced electron transfer, foretelling a bright future for this technique.

## Methods

### Large-scale cell culture of *T. elongatus*

Large-scale cultures of *T. elongatus* were grown in a 120-L cultivation photobioreactor (Photon System Instruments) that features controllable light intensity, timing, and modulation (Supplementary Fig. 1a). The starter cultures were maintained in 1-L flasks, and cells were grown in autoclaved BG-11 medium with constant agitation at 56 °C by using New Brunswick Innova shaker incubators. The small starter cultures were used to inoculate a 25-L photobioreactor (Photon System Instruments) that features the same control system as the 120-L reactor. Cell growth was monitored daily, and when the absorbance at 750 nm (A_750_) reached 0.4, the culture was used to inoculate the 120-L photobioreactor. Initially, the cultures were dark-adapted for 6 h, and then the light intensity was linearly increased to 200 µE (red:white = 3:1). The culture was harvested (in 4–5 days) when A_750_ reached 0.8.

For cell harvesting, the entire volume of the photobioreactor (120 L) was collected in a large container. The first stage of concentration was performed by using Cole Parmer’s Masterflex Peristaltic Pump tangential flow filter system (Cat# 200-1558). Flow-through was discarded while the cells were concentrated in the filter with each flow cycle. The cell suspension was collected in a fresh beaker by flushing the system with purified water. The culture volume was reduced to 2–2.5 L, and the collected cells were centrifuged at 7000×*g* at RT. After the first centrifugation round, additional cell suspension was added on top of the initial pellet and sedimented until all cells were separated from the supernatant. Cell pellets were frozen and stored at −80 °C. A typical harvest yielded ~80 g of cells per 120 L of cell culture.

### PSI purification

Cells were thawed, and PSI was isolated as previously described with modifications^28^. All steps were performed in dim green light. Cells were resuspended in buffer A (20 mM MES, pH = 6.4, 10 mM CaCl_2_, and 10 mM MgCl_2_) and centrifuged at 4 °C at 7400×*g* to wash cells before being resuspended in lysis buffer (20 mM MES, pH = 6.4, 10 mM CaCl_2_, 10 mM MgCl_2_, and 500 mM mannitol) and subsequently lysed by using a microfluidizer (Microfluidics Model M110-L) at 12,000 psi on ice. The cell lysate was sequentially centrifuged, and the thylakoid membranes were washed at 4 °C four times before the final resuspension in buffer A. Protein concentration was determined by performing chlorophyll (Chl) assays in 80% acetone (ɛ_664_ = 76780 M^−1^ cm^−1^). PSI was solubilized in buffer A with 0.75% weight per volume (w/v) β-dodecylmaltoside (β-DDM) for 45 min at a final Chl concentration of 0.75 mM. This solubilization extract was centrifuged for 2 h at 235,000 × *g* (Ti-45, Beckman Coulter) at 4 °C. The top layer of the supernatant (~10–15 mL/tube) was removed and discarded. The remaining supernatant was gently shaken in an orbital shaker at 4 °C for 45 min to separate PSI from the top layer of the membrane pellet. A Chl assay was performed on the supernatant, and aliquots equivalent to 80 µmol of Chl were injected onto an XK 50/60 column (GE Healthcare) packed with DEAE anion exchange resin (Toyopearl) with a bed height of ~40 cm equilibrated with 20 mM MES, pH = 6.4, 15 mM MgSO_4_, and 0.02% w/v β-DDM. A step gradient was applied to elute first the PSI and PSII monomers and PSII dimers, before the PSI trimers were eluted from the column by a step gradient increasing the MgSO_4_ concentration to 150 mM. PSI eluted at a MgSO_4_ concentration of 145 mM (Supplementary Fig. 1b). The PSI fractions were pooled from multiple runs and concentrated in an Amicon 400-mL stirred ultrafiltration cell (EMD Millipore) fitted with a 100-kDa cutoff filter (Millipore) by using a headspace pressure of 30–60 psi argon. Once the target concentration of 10 mM Chl had been reached (typically ~100-fold concentration of the initial volume), the concentrated solution was diluted dropwise by addition of buffer G_0_ (5 mM MES, pH = 6.4, 0.02% w/v β-DDM) to achieve a final MgSO_4_ concentration of 4 mM. This solution was concentrated to 10 mM Chl. The following day, ~10 mL of buffer G_0_ was applied to the mat of crystallized PSI that had formed on the membrane, and gentle pipette mixing was performed to harvest the crystals and remove them from the filter. These crystals were shipped to the EuXFEL as a crystal suspension at 4 °C and recrystallized on-site at the XFEL Biology Infrastructure (XBI) User Consortium laboratory, as crystal quality decreases during transport.

The crystal-containing aliquots could be pooled and sequentially settled for 10, 20, and 30 min, and overnight as described by Hunter^18^ to sort the crystals by size. For this beamtime, the small crystals were separated from the larger crystals in one settling step overnight. The supernatant of the overnight settling step contained small uniform nanocrystals, which were saved as a separate sample to be used for seeding. The crystals were stored in buffer G_0_ at 4 °C. The seeds were characterized by DLS (Fig. 1b) to determine size and homogeneity profiles of the nanocrystal seeds. We also periodically performed SONICC analysis^44^ to verify the crystallinity of the seed stock suspensions. All samples were stored at 4 °C in dim green light until further use in the final recrystallization experiments directly prior to the XFEL data collection.

### Recrystallization

All crystals used for data collection were freshly grown at the XBI User Consortium laboratory at the EuXFEL directly prior to the experiment, which ensured size homogeneity and avoided damage during transport. The first fine screening was performed in test batches for each individual protein preparation prior to crystallization in larger sample-scale batches suitable for use in the SFX experiments. PSI was crystallized by decreasing the ionic strength with nanocrystal seeds added to the precipitant buffer (Supplementary Fig. 3). For each crystallization experiment, the pooled larger PSI crystals were dissolved by addition of MgSO_4_ and subsequently recrystallized. A harvested preparation of PSI crystals was homogeneously resuspended by gentle pipette mixing before an aliquot was transferred to a preweighed microcentrifuge tube. This aliquot was then centrifuged at 13,000×*g* for 2 min followed by removal of the bulk supernatant. This was repeated twice, and the final portion of supernatant was removed before weighing the crystal pellet. A µL:mg equivalent of buffer G_100_ (100 mM MgSO_4_, 5 mM MES, pH = 6.4, and 0.02% w/v β-DDM) was added to the crystal pellet via pipette mixing, and the crystals were allowed to dissolve for at least 30 min at RT. Prior to crystallization experiments, optical microscopy by using a polarized filter was used to confirm that the solution was homogeneous and all crystals were dissolved. A Chl assay was then performed followed by dilution to 28 mM Chl (100 mg PSI/mL) by using buffer G_50_ (50 mM MgSO_4_, 5 mM MES, pH = 6.4, and 0.02% w/v β-DDM). To determine the optimal crystallization conditions, 1 µL of this solution was placed in a 1.5-mL reaction vessel. The precipitant buffers G_X_, were prepared, which contained no (G_0_), 1 (G_1_), 2 (G_2_), or 3 mM MgSO_4_ (G_3_) in 20 mM MES, pH = 6.4, 0.02% w/v β-DDM. Thirty seconds prior to mixing with the concentrated PSI, nanocrystal seeds were added to the G_X_ solution ([Chl] = 3 µM) to achieve homogeneous nucleation. The final concentration was 487 µM Chl. These test batches were then allowed to crystallize for 1–2 h before visual inspection with optical microscopy with emphasis on optimization of apparent morphology, size, and homogeneity. Small batch screening showed a large effect of MgSO_4_ concentration on the size, size homogeneity, and morphology of the crystals as illustrated in Supplementary Fig. 2. Crystal macromorphology also served as an indicator; visible defects appeared more frequently at lower salt concentrations, possibly attributable to higher supersaturation conditions leading to faster crystal growth where crystallogenic growth kinetics outpace the individual molecular Gibbs energy minimization during lattice addition.

The crystal growth was typically complete in these test batches within 1–2 h, but interpretable results were realized within 30 min with no appreciable difference in ranking characteristics occurring thereafter. Supplementary Fig. 2 shows an example of the results from a crystallization test set. Once a suitable condition had been identified, scaled-up crystallogenesis was performed to obtain the larger volumes necessary for MHz crystallography as described in the “Results” section. The large-scale crystallization was achieved by using the RAMS method described in detail in Supplementary Fig. 3. Here, the PSI crystals were dissolved and diluted to 28 mM Chl as described above for the small test crystallization experiments. This concentrated solution was then added dropwise to the base of a suitably large beaker to allow for adequate surface area such that 5–20-µL drops could be spread distinctly without consolidation. The previously optimal G_X_ solution was then doped with the same seed batch used during optimization and added on top of the protein drops at once to reach a Chl concentration of 487 µM Chl while simultaneous rotational mechanical mixing was performed (~120–180 rpm). Rotation was continued for at least 30 s beyond homogenization by eye. The solution was then transferred to a 50-mL conical tube and allowed to settle at RT overnight before characterization by light microscopy in both bright-field and under polarized light to check for signature birefringence. Upscaling from test batches to the large-scale RAMS method showed a general improvement in size homogeneity, possibly attributable to more rapid complete mixing, illustrated by homogeneous rod-shaped crystals depicted in Fig. 1c.

### Sample delivery

In preparation for sample delivery, PSI microcrystals were allowed to settle by gravity overnight at 4 °C. The volume of the settled crystals was approximated, and most of the supernatant was removed to leave a volume of supernatant equal to the volume of the settled crystals. The crystals were then gently resuspended in the supernatant, and the crystal suspension was prefiltered through the same 20-µm stainless-steel filter (IDEX) that was used for inline filtering during sample delivery. The sample reservoirs were mounted on an anti-settling device that maintained the temperature of the crystals at 4 °C at the SBP/SFX instrument. The anti-settling device was developed by Robert Shoeman at the MPI Heidelberg^30^. The crystals were delivered to the XFEL beam by using a GDVN liquid injection system^12,13^ with 50-µm inner-diameter glass capillaries that were individually hand-ground and tested at Arizona State University for the production of a straight jet and high sample flow rates. The small ID of the nozzle compared with the crystal size could have led to significant challenges for sample delivery. Our previous experience at XFELs indicated that ideal crystals should be maximally 1/10th the size of the ID of the nozzle to avoid clogging. Based on the size of our crystals (5 × 5 × 15 µm^3^), 100-µm-ID nozzles could have been used. However, even when the flow rate is increased to 30 µL/min, a jet speed of only ~12.5 m/s is achieved with this nozzle, which is too slow to fully replenish the sample between pulses. The sample was delivered at a flow rate of 20 µL/min to the injector by use of a syringe-reservoir system^30^ where pressurized water from a high-performance liquid chromatography (HPLC) pump (Shimadzu) was delivered to the back side of the sample reservoir that displaces a fitted Teflon plunger separating the loaded crystal suspension from the pressurizing water. The liquid stream was focused by the sheath jet of co-propagating helium gas in the GDVN resulting in a jet speed of 50 m/s^11^. The gas flow was controlled by using a GP1 gas-pressure regulator (Proportion-air), and the flow rate was monitored with a gas flow meter (Bronkhorst). Required jet speeds were estimated based on the recovery of the jet as described by Wiedorn et al.^11^.

### Data collection

The PSI SFX experiments were performed by using the SPB/SFX instrument at the EuXFEL in November 2017 during the experiment P2066^20^ in a similar manner to Wiedorn et al.^11^. Data were collected at a photon energy of 9.3 keV with an average XFEL pulse energy of 0.7–1 mJ, and pulse duration of 50 fs. We estimate that the beam focus diameter was 16 ± 4 μm (FWHM) based on the optical imaging of single shots by using Ce:YAG screens of various thicknesses. The X-ray diffraction data were recorded in single-shot mode by using the AGIPD 1 Mpixel with the direct beam passing through a central hole in the detector. The AGIPD consists of 2 × 8 application-specific integrated circuits, each with 128 × 512 pixels of 200 × 200- µm size^45^ and allows the collection of data at a frame rate matching the EuXFEL pulse rate of 1.1–4.5 MHz within a pulse train. This enables the measurement of up to 3520 images per second (in the 352 memory cells of each pixel per pulse train). Each pixel automatically switches between three gain modes from most to least sensitive: high, medium, and low, which allows for data collection with high dynamic range (~1 × 10^4^ photons at 12 keV). For more details about the detector, the reader is referred to Allahgholi et al.^45^.

### Data analysis

Data from each AGIPD module were saved into separate files along with pulse and train ID numbers. The EuXFEL version of the hit-finding program Cheetah^23^, as described in Wiedorn et al.^11^, was used to match data from each of the 16 separate modules by train and pulse number, to process and compare data from the same X-ray pulse. Calibration of the AGIPD readout requires measurement of the pedestal, gain, and gain-switching threshold for each of the three gain stages in each memory cell of each pixel. AGIPD calibration, multi-gain-stage intensity correction, and masking of bad memory cells for each pixel were performed as described in Wiedorn et al.^11^. Although the detector required complicated calibration^11^, this step was essential to obtain more accurate intensities simultaneously for both the high- and low-resolution reflections, all of which are only partially measured in each snapshot.

The initial detector geometry was taken from the previous experiment^11^ and further refined by using *geoptimiser* program^37^. We used the three-ring integration method in *indexmajig*^24,25^, in which the outer annulus around each peak is used to calculate the local background and sigma. Integration radii were 2, 3, and 6 pixels to account for the small spot spacing. The actual sample-to-detector distance for different detector positions was determined by the following criteria: at the correct detector distance the unit cell distribution has to be symmetrical. Small residual asymmetry (Fig. 2a) is probably due to the small shot-to-shot variation of the incident beam wavelength. The detector center was adjusted individually for the two detector positions due to the detector stage being slightly misaligned from the optical axis.

Hit finding was performed with Cheetah^23^ by using the peakfinder8 algorithm with conservative parameters: minimum signal-to-noise ratio (SNR of 6, pixel threshold of 200, minimum of 3 pixels per peak, and a minimum of 20 peaks per pattern). For the indexing (using *indexmajig* from CrystFEL version 0.8.0 + 1ccb8c35), different peak-finding parameters were used: minimum SNR of 6, pixel threshold of 50, minimum pixels per peak = 1, and a minimum of 50 peaks per pattern. To find peaks that are only one pixel in size, careful masking of unreliable regions on the detector was performed. In total (for all 30 pulses per train) 54,780 of 59,012 patterns were indexed. Of these, 99.8% of the 54,780 patterns were indexed by using *Xgandalf*^26^ and the remaining 0.2% were indexed by using MOSFLM^46^ and DirAx^47^. The resulting unit cell distributions are very narrow with a clearly monoclinic (pseudohexagonal) lattice (Fig. 2a). Xgandalf^26^ is a recently available indexing algorithm that demonstrated outstanding results, but so far only a few experiments had employed it. Therefore, to verify the unit cell parameters, the indexing was repeated by using only MOSFLM^46^ with the same CrystFEL parameters. This check resulted in very similar cell parameters (Supplementary Fig. 12) but with a wider, bimodal distribution for the *a*-axis. Also, indexing by using only MOSFLM resulted in fewer indexed patterns: 39,985 of 59,012.

Multicrystal indexing was not used in *indexamajig* because of the density of reflections from individual PSI microcrystals. The very small Bragg reflection profile radii (calculated by *indexamajig* to account for the large majority of the found reflections) are evidence of the high accuracy of indexing results from *Xgandalf* and orientation refinement in *indexamajig* (shown in Supplementary Fig. 6a–c for each detector distance). Supplementary Fig. 6d shows the diffraction resolution limits for all 30 pulses in the train, showing no evidence of sample damage in consecutive pulses. Data collection statistics for the XFEL data were calculated by using CrystFEL^25^ and for the synchrotron data using Aimless in the CCP4 software suite^42^ (Table 2). The reflections from the first ten pulses (the dark structure) were merged with *process_hkl* in point group 2 /m. The resulting Rsplit, CC*, the SNR, and completeness are shown in Supplementary Fig. 5.

### Structure solution and refinement

For both the RT XFEL structure and cryogenic synchrotron structure, the phases were determined by molecular replacement with Phenix’s implementation of Phaser^27^ by using the previously determined structure of PSI from *T. elongatus*^28^ as a starting model. All water molecules were removed from the starting model. R_free_ flags were assigned (5%) with phenix.refine^48^. Three rounds of refinement by using phenix.refine^48^, three rounds of refinement by using REFMAC5^49^, and one round of find:waters by using Coot^50^ were run on each data set by using the corresponding molecular replacement solution from Phaser. Refinement statistics were calculated with Phenix^51^. For data quality assessment of the RT XFEL structure in addition to the standard refinement output statistics, an annealing composite omit map omitting 0.5% of the atoms of the model within the asymmetric unit was generated by using the corresponding function within the Phenix software suite^51^ and is shown in Supplementary Fig. 13. The simulated annealing omit map appears similar to the 2Fo-Fc map (Fig. 3). We have also performed manual omission of various ligands in the XFEL structure and re-refined the map with the incomplete model. Clear density can be seen where the omitted ligand was removed (Supplementary Fig. 14).

### Reporting summary

Further information on research design is available in the Nature Research Reporting Summary linked to this article.

## Supplementary information

Supplementary Information

Reporting Summary

## Data Availability

The source data underlying Fig. 2c and Supplementary Fig. 5 are provided as a Source Data File. Other data are available from the corresponding authors upon request. The XFEL structure has been deposited with the PDB accession code 6PGK, and the synchrotron structure has been deposited with the PDB accession code 6PFY. XFEL-collected diffraction data have been deposited in the Coherent X-ray Imaging Data Bank (CXIDB) under accession code 111.

## Source data

Source Data

## Code availability

The versions of Cheetah and CrystFEL used in this work are available from the respective websites: https://www.desy/de/~barty/cheetah and https://www.desy.de/~twhite/crystfel. The Xgandalf software is available at https://stash.desy.de/users/gevorkov/repos/xgandalf and is now implemented in the CrystFEL version 8.0 software suite.

## References

[1] Boutet S (2012). High-resolution protein structure determination by serial femtosecond crystallography. Science.

[2] Chapman HN (2011). Femtosecond X-ray protein nanocrystallography. Nature.

[3] Aquila A (2012). Time-resolved protein nanocrystallography using an X-ray free-electron laser. Opt. Express.

[4] Tenboer J (2014). Time-resolved serial crystallography captures high-resolution intermediates of photoactive yellow protein. Science.

[5] Stagno JR (2017). Structures of riboswitch RNA reaction states by mix-and-inject XFEL serial crystallography. Nature.

[6] Kupitz C (2017). Structural enzymology using X-ray free electron lasers. Struct. Dyn..

[7] Olmos JL (2018). Enzyme intermediates captured ‘on the fly’ by mix-and-inject serial crystallography. BMC Biol..

[8] Barends TRM (2015). Direct observation of ultrafast collective motions in CO myoglobin upon ligand dissociation. Science.

[9] Schmidt M (2019). Time-resolved macromolecular crystallography at pulsed X-ray sources. Int. J. Mol. Sci..

[10] Grünbein ML (2018). Megahertz data collection from protein microcrystals at an X-ray free-electron laser. Nat. Commun..

[11] Wiedorn MO (2018). Megahertz serial crystallography. Nat. Commun..

[12] DePonte DP (2008). Gas dynamic virtual nozzle for generation of microscopic droplet streams. J. Phys. D. Appl. Phys..

[13] Wiedorn MO (2018). Rapid sample delivery for megahertz serial crystallography at X-ray FELs. IUCrJ.

[14] Galayda, J. N. The new LCLS-II project: status and challenges. in *LINAC2014* 404–408 (2014). https://pdfs.semanticscholar.org/4c4f/51c8414d37fae9bd3cbb70f53da2d599fc14.pdf.

[15] Henrich B (2011). The adaptive gain integrating pixel detector AGIPD a detector for the European XFEL. Nucl. Instrum. Methods Phys. Res. Sect. A Accel. Spectrometers, Detect. Assoc. Equip..

[16] Allahgholi A (2019). Megapixels @ Megahertz – the AGIPD high-speed cameras for the European XFEL. Nucl. Instrum. Methods Phys. Res. Sect. A Accel. Spectrometers, Detect. Assoc. Equip..

[17] Yang Huixia, Liu Jun, Wen Xiaogang, Lu Congming (2015). Molecular mechanism of photosystem I assembly in oxygenic organisms. Biochimica et Biophysica Acta (BBA) - Bioenergetics.

[18] Hunter MS, Fromme P (2011). Toward structure determination using membrane-protein nanocrystals and microcrystals. Methods.

[19] Altarelli, M. et al. *The European X-Ray Free-Electron Laser–Technical Design Report* (2007). https://xfel.desy.de/localfsExplorer_read?currentPath=/afs/desy.de/group/xfel/wof/EPT/TDR/XFEL-TDR-final.pdf.

[20] Mancuso AP (2019). The single particles, clusters and biomolecules and serial femtosecond crystallography instrument of the European XFEL: initial installation. J. Synchrotron Radiat..

[21] Fangohr, H. et al. Data analysis support in Karabo at European XFEL. In *16th International Conference on Accelerator and Large Experimental Control Systems,* TUCPA01, 245–252 (Barcelona, Spain, 2017).

[22] Mariani V (2016). OnDA: online data analysis and feedback for serial X-ray imaging. J. Appl. Cryst..

[23] Barty A (2014). Cheetah: software for high-throughput reduction and analysis of serial femtosecond X-ray diffraction data. J. Appl. Cryst..

[24] White TA (2016). Recent developments in CrystFEL. J. Appl. Crystallogr..

[25] White TA (2012). CrystFEL: a software suite for snapshot serial crystallography. J. Appl. Crystallogr..

[26] Gevorkov Yaroslav, Yefanov Oleksandr, Barty Anton, White Thomas A., Mariani Valerio, Brehm Wolfgang, Tolstikova Aleksandra, Grigat Rolf-Rainer, Chapman Henry N. (2019). XGANDALF – extended gradient descent algorithm for lattice finding. Acta Crystallographica Section A Foundations and Advances.

[27] McCoy AJ (2007). Phaser crystallographic software. J. Appl. Crystallogr..

[28] Jordan P (2001). Three-dimensional structure of cyanobacterial Photosystem I at 2.5 Å resolution. Nature.

[29] Fromme P, Witt HT (1998). Improved isolation and crystallization of photosystem I for structural analysis. Biochim. Biophys. Acta - Bioenerg..

[30] Lomb L (2012). An anti-settling sample delivery instrument for serial femtosecond crystallography. J. Appl. Crystallogr..

[31] Grünbein, M. L. & Nass Kovacs, G. Sample delivery for serial crystallography at free-electron lasers and synchrotrons. *Acta Crystallogr. Sect. D* **75**, 178–191 (2019). 10.1107/S205979831801567X PMC6400261 30821706

[32] Weierstall Uwe (2014). Liquid sample delivery techniques for serial femtosecond crystallography. Philosophical Transactions of the Royal Society B: Biological Sciences.

[33] Oberthuer D (2017). Double-flow focused liquid injector for efficient serial femtosecond crystallography. Sci. Rep..

[34] Weierstall U (2014). Lipidic cubic phase injector facilitates membrane protein serial femtosecond crystallography. Nat. Commun..

[35] Echelmeier Austin, Kim Daihyun, Cruz Villarreal Jorvani, Coe Jesse, Quintana Sebastian, Brehm Gerrit, Egatz-Gomez Ana, Nazari Reza, Sierra Raymond G., Koglin Jason E., Batyuk Alexander, Hunter Mark S., Boutet Sébastien, Zatsepin Nadia, Kirian Richard A., Grant Thomas D., Fromme Petra, Ros Alexandra (2019). 3D printed droplet generation devices for serial femtosecond crystallography enabled by surface coating. Journal of Applied Crystallography.

[36] Kim D (2019). Electric triggering for enhanced control of droplet generation. Anal. Chem..

[37] Yefanov O (2015). Accurate determination of segmented X-ray detector geometry. Opt. Express.

[38] Krauss N (1993). 3-dimensional structure of system-I of photosynthesis at 6 angstrom resolution. Nature.

[39] Klukas O (1999). Localization of two phylloquinones, QK and QK′, in an improved electron density map of Photosystem I at 4-Å resolution. J. Biol. Chem..

[40] Klukas O (1999). Photosystem I, an improved model of the stromal subunits PsaC, PsaD, and PsaE. J. Biol. Chem..

[41] Krauß N (1996). Photosystem I at 4 Å resolution represents the first structural model of a joint otosynthetic reaction centre and core antenna system. Nat. Struct. Biol..

[42] Winn MD (2011). Overview of the CCP4 suite and current developments. Acta Crystallogr. Sect. D. Biol. Crystallogr..

[43] Malavath T, Caspy I, Netzer-El SY, Klaiman D, Nelson N (2018). Structure and function of wild-type and subunit-depleted photosystem I in *Synechocystis*. Biochim. Biophys. Acta - Bioenerg..

[44] Wampler RD (2008). Selective detection of protein crystals by second harmonic microscopy. J. Am. Chem. Soc..

[45] Allahgholi A (2019). The adaptive gain integrating pixel detector at the European XFEL. J. Synchrotron Radiat..

[46] Battye TGG, Kontogiannis L, Johnson O, Powell HR, Leslie AGW (2011). IMOSFLM: a new graphical interface for diffraction-image processing with MOSFLM. Acta Crystallogr. Sect. D..

[47] Duisenberg AJM (1992). Indexing in single-crystal diffractometry with an obstinate list of reflections. J. Appl. Crystallogr..

[48] Afonine PV (2012). Towards automated crystallographic structure refinement with phenix.refine. Acta Crystallogr. Sect. D..

[49] Murshudov GN (2011). REFMAC5 for the refinement of macromolecular crystal structures. Crystallogr. Sect. D..

[50] Emsley P, Lohkamp B, Scott WG, Cowtan K (2010). Features and development of Coot. Acta Crystallogr. Sect. D. Biol. Crystallogr..

[51] Adams PD (2010). PHENIX: A comprehensive python-based system for macromolecular structure solution. Acta Crystallogr. Sect. D. Biol. Crystallogr..

