# Author Correction: Membrane protein megahertz crystallography at the European XFEL

**DOI:** 10.1038/s41467-020-14436-4

**Published:** 2020-01-30

**Authors:** Chris Gisriel, Jesse Coe, Romain Letrun, Oleksandr M. Yefanov, Cesar Luna-Chavez, Natasha E. Stander, Stella Lisova, Valerio Mariani, Manuela Kuhn, Steve Aplin, Thomas D. Grant, Katerina Dörner, Tokushi Sato, Austin Echelmeier, Jorvani Cruz Villarreal, Mark S. Hunter, Max O. Wiedorn, Juraj Knoska, Victoria Mazalova, Shatabdi Roy-Chowdhury, Jay-How Yang, Alex Jones, Richard Bean, Johan Bielecki, Yoonhee Kim, Grant Mills, Britta Weinhausen, Jose D. Meza, Nasser Al-Qudami, Saša Bajt, Gerrit Brehm, Sabine Botha, Djelloul Boukhelef, Sandor Brockhauser, Barry D. Bruce, Matthew A. Coleman, Cyril Danilevski, Erin Discianno, Zachary Dobson, Hans Fangohr, Jose M. Martin-Garcia, Yaroslav Gevorkov, Steffen Hauf, Ahmad Hosseinizadeh, Friederike Januschek, Gihan K. Ketawala, Christopher Kupitz, Luis Maia, Maurizio Manetti, Marc Messerschmidt, Thomas Michelat, Jyotirmoy Mondal, Abbas Ourmazd, Gianpietro Previtali, Iosifina Sarrou, Silvan Schön, Peter Schwander, Megan L. Shelby, Alessandro Silenzi, Jolanta Sztuk-Dambietz, Janusz Szuba, Monica Turcato, Thomas A. White, Krzysztof Wrona, Chen Xu, Mohamed H. Abdellatif, James D. Zook, John C. H. Spence, Henry N. Chapman, Anton Barty, Richard A. Kirian, Matthias Frank, Alexandra Ros, Marius Schmidt, Raimund Fromme, Adrian P. Mancuso, Petra Fromme, Nadia A. Zatsepin

**Affiliations:** 1 Biodesign Center for Applied Structural Discovery, Arizona State University, Tempe, AZ 85287-5001 USA; 2 School of Molecular Sciences, Arizona State University, Tempe, AZ 85287-1604 USA; 3 European XFEL GmbH, Holzkoppel 4, 22869 Schenefeld, Germany; 4 Center for Free-Electron Laser Science, Deutsches Elektronen-Synchrotron, Notkestrasse 85, 22607 Hamburg, Germany; 5 Department of Physics, Arizona State University, Tempe, AZ 85287-1504 USA; 6 Hauptman-Woodward Institute, 700 Ellicott St, Buffalo, NY 14203-1102 USA; 7 Department of Structural Biology, Jacobs School of Medicine and Biomedical Sciences, SUNY University at Buffalo, 700 Ellicott St, Buffalo, NY 14203-1102 USA; 8 Linac Coherent Light Source, SLAC National Accelerator Laboratory, Menlo Park, 94025 CA USA; 9 Department of Physics, Universität Hamburg, Luruper Chaussee 149, 22761 Hamburg, Germany; 10 The Hamburg Centre for Ultrafast Imaging, Universität Hamburg, Luruper Chaussee 149, 22761 Hamburg, Germany; 11 Deutsches Elektronen-Synchrotron, Notkestrasse 85, 22607 Hamburg, Germany; 12 Institute for X-Ray Physics, University of Göttingen, 37077 Göttingen, Germany; 13 Center Nanoscale Microscopy and Molecular Physiology of the Brain, Göttingen, Germany; 14 Biological Research Centre, Hungarian Academy of Sciences, Temesvári krt. 62, Szeged, 6726 Hungary; 15 Department of Biochemistry & Cellular and Molecular Biology, University of Tennessee at Knoxville, Knoxville, TN USA 37996; 16 Program in Energy Science and Engineering, University of Tennessee at Knoxville, Knoxville, TN USA 37996; 17 Department of Microbiology, University of Tennessee at Knoxville, Knoxville, TN USA 37996; 18 Lawrence Livermore National Laboratory, 7000 East Avenue, Livermore, CA 94550 USA; 19 University of Southampton, University Rd, Southampton, SO17 1BJ UK; 20 Hamburg University of Technology, Vision Systems E-2, Harburger Schloßstraße 20, 21079 Hamburg, Germany; 21 Department of Physics, University of Wisconsin-Milwaukee, 3135 N. Maryland Ave, Milwaukee, WI 53211 USA; 22 Department of Chemistry and Physics, La Trobe Institute for Molecular Science, La Trobe University, Melbourne, 3086 Victoria Australia; 23 Present Address: Department of Chemistry, Yale University, New Haven, CT 06520 USA; 24 Present Address: Deutsches Elektronen-Synchrotron, Notkestrasse 85, 22607 Hamburg, Germany; 25 Present Address: ARC Centre of Excellence in Advanced Molecular Imaging, Department of Chemistry and Physics, La Trobe Institute for Molecular Science, La Trobe University, Melbourne, 3086 Victoria Australia

**Keywords:** X-ray crystallography, X-ray crystallography, Nanocrystallography, Nanocrystallography, Structural biology

Correction to: *Nature Communications* 10.1038/s41467-019-12955-3, published online 4 November 2019.

The original version of this Article contained errors in the email address of the corresponding author, Nadia A. Zatsepin, as well in her current affiliation. These have now been corrected in both the PDF and HTML versions of the article.

