# Supplementary material for: Membrane protein megahertz crystallography at the European XFEL
Source: Nat Commun. 2019 Nov 4;10:5021. doi: 10.1038/s41467-019-12955-3 (PMC6828683; doi:10.1038/s41467-019-12955-3)
Supplement: Supplementary file 1 — Supplementary Information [file 41467_2019_12955_MOESM1_ESM.pdf]

Supplementary Information

Membrane Protein Megahertz Crystallography at the European XFEL

Christopher Gisriel, Jesse Coe et al.

Supplemental Notes:

Supplementary Note 1. Crystallization, data collection, data analysis, and structure solution for the cryogenic synchrotron structure of *T. elongatus* PSI presented here.

Crystallization. For the PSI crystallized for synchrotron X-ray diffraction at cryogenic conditions, crystallization was performed as described previously[1] with further details described in Fromme 1998[2] . Briefly, the crystals were grown at low ionic strength by dialysis with micro- and subsequent macro-seeding steps. The freezing procedure also matched the procedure used for the Jordan et al. PSI structure (PDB ID=1JB0)[1]. Crystals were transferred in 10 steps from the crystal stabilization buffer (5 mM MES pH=6.4, 0.02% β-DDM) into the freezing buffer (2 M sucrose, 5 mM MES pH=6.4, 0.02% β-DDM) and incubated for 1 hour in the 2 M sucrose buffer before crystals were fished into cryo-loops and flash-frozen in liquid propane.

Data collection and analysis. Data were collected at the Advanced Light Source (ALS) at Lawrence Berkeley National Laboratory at beamline 8.2.1 at 100 K with a 100 x 200 µm beam focus. The data was collected from a single crystal which was rotated one degree per image and 180 images were collected. The data set was indexed and merged in XDS[3]. The space group determination and scaling was achieved with Aimless from the CCP4 software suite[4].

Supplementary Note 2. Crystallization at low ionic strength for SFX and standard crystallography.

Crystallization at low ionic strength offers potential to perform SFX studies of other difficult to crystallize proteins at MHz repetition rates. Most proteins are less soluble at low ionic strength compared to medium ionic strength. At low ionic strength protein interactions are enabled by hydrophilic interactions. As the surface of the protein is depleted of counter ions that effectively shield opposite charges on the surface of a protein, direct contacts between opposing surface charges induce the formation of crystal contacts. In contrast, crystallization at the other side of the phase diagram is induced when the solubility of the protein is decreased by addition of crystallization agents like PEG or at high ionic strength. Here, the protein and ions or PEG compete for the water needed for solvation. Thereby at high ionic strength or in the presence of PEG, hydrophobic interactions are enhanced. While crystallization at low ionic strength is not a novel method of crystallization[5], it is rarely used for crystallization of membrane proteins. This is most likely a matter of convenience; vapor diffusion is the most common method for crystallization and is the basis of almost all robotic crystallization systems. While crystallization at low ionic strength is quite labor-intensive for standard crystallography, it is the a very useful method for growth of nano- and microcrystals for MHz crystallography. In addition to the advantages for SFX listed in the main text it is also easy to perform and is reproducible. Crystals form quickly by ultrafiltration crystallization, can be used as a final purification step and the method is also fully reversible (no “PEG skin” is formed that can prevent seeding when PEG is used for crystallization). Thereby, seeding is easily implemented and the protein can be crystallized and dissolved many times to obtain the desired crystal size and purity.

Supplementary Tables:

Supplementary Table 1 Summary of various XFEL start-dates, pulse delivery, maximum frequency, and minimum time between pulses

	Start of user operation	Pulse delivery	Maximum pulses per second	Minimum time between pulses (ms)
LCLS[1#]	2009	constant frequency	120	8.33
SACLA[2#]	2011	constant frequency	60	16.67
PAL-XFEL[3#]	2016	constant frequency	30	16.67
EuXFEL[4#*]	2017	pulse trains at 10 Hz	2,500[#]<br>27,000*	0.000886[#]<br>0.000222*
SwissFEL[5#]	2018	constant frequency	100	10
LCLS-II[6*]	2021*	constant frequency	1000000*	0.001

[#]Current operational capability.

*Proposed operational capability.

The AGIPD[6,7] will be able to store 3520 diffraction patterns per second.

Supplementary Table 2 Comparison of PSI structures determined using crystallographic data collected at the EuXFEL and at the Advanced Photon Source synchrotron. Various superpositions were performed using PyMOL[8] and their RMSD values are shown. The two structures were similar at 2.9 Å resolution, especially when comparing individual subunits. For reference, the poly-Ala superposition of two evolutionarily closely-related polypeptides, PsaA and PsaB, is ~0.8 Å and the superposition of the poly-Ala chains of the two single-transmembrane helix (TMH) subunits PsaI and PsaJ is ~4.3 Å. M1 corresponds to the PSI monomer whose core polypeptides are chains A and B in the PDB file, M2 corresponds to the PSI monomer whose core polypeptides are chains G and H in the PDB file, and M3 corresponds to the PSI monomer whose core polypeptides are chains Y and Z in the PDB file.

**Complete structure comparison**	**RMSD of poly-Ala chains only (Å)**	**RMSD of full structures (Å)**
	0.293	0.318

**Comparison by protein subunit RMSDs by subunit poly-Ala chains only (Å)**			
	**M1**	**M2**	**M3**
**PsaA**	0.246	0.254	0.262
**PsaB**	0.245	0.253	0.244
**PsaC**	0.237	0.261	0.245
**PsaD**	0.272	0.243	0.268
**PsaE**	0.228	0.248	0.219
**PsaF**	0.246	0.272	0.257
**PsaI**	0.231	0.251	0.251
**PsaJ**	0.214	0.211	0.236
**PsaK**	0.270	0.235	0.277
**PsaL**	0.282	0.272	0.254
**PsaM**	0.230	0.204	0.235
**PsaX**	0.220	0.225	0.222

**Comparison by protein subunit RMSD by subunit full chains**

	M1	M2	M3
**PsaA**	0.272	0.279	0.287
**PsaB**	0.274	0.280	0.271
**PsaC**	0.272	0.286	0.267
**PsaD**	0.307	0.281	0.311
**PsaE**	0.261	0.289	0.258
**PsaF**	0.273	0.308	0.291
**PsaI**	0.252	0.266	0.278
**PsaJ**	0.239	0.291	0.272
**PsaK**	0.307	0.272	0.279
**PsaL**	0.298	0.294	0.290
**PsaM**	0.265	0.245	0.269
**PsaX**	0.231	0.239	0.239

Supplementary Table 3 Comparison of molecular weight, unit cell dimensions, crystal size, and approximate number of unit cells per crystal between the PSI structure determined here and previous structures determined by MHz crystallography.

Molecule name	Molecular weight (kDa)	Unit cell dimensions (a, b, c (Å), α, β, γ)	Quoted crystal size (μm)	Approx. crystal volume (μm^3)	Approx. unit cells x 10^6 / crystal
Photosystem I	1036.1	279.2, 164.5, 284.1, 90, 119.30, 90	5x5x15	375	33
Lysozyme	14.9	79.6, 79.6, 38.3, 90, 90, 90	6-8	343	1413
β-lactamase	27.9	41.8, 41.8, 233.3, 90, 90, 120	3-8	166	471
Concanavalin A	25.7	63.9, 88.1, 90.2, 90, 90, 90	5-10	422	831
Concanavalin B	33.9	82.3, 82.3, 103.4, 90, 90, 120	5-10	422	696

Supplementary Figures:

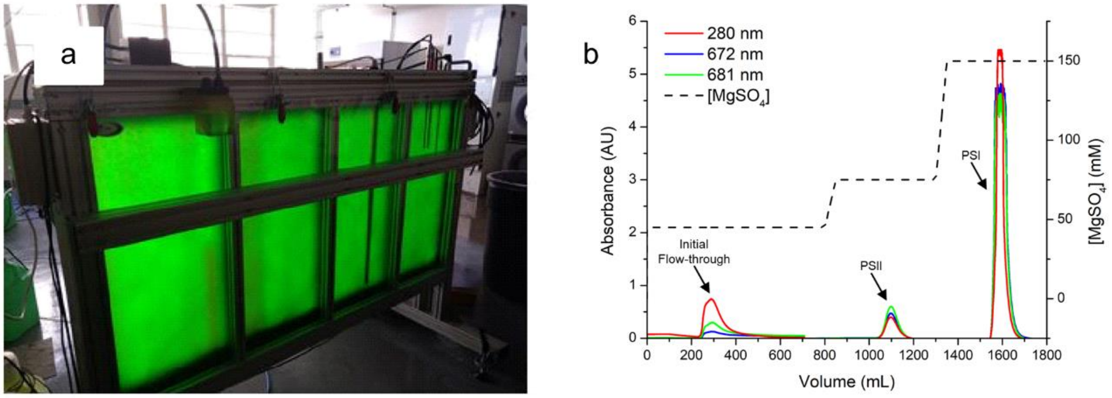

Supplementary Fig. 1 Cell growth and chromatography for PSI isolation. **a** 120 L photobioreactor used for growth of the thermophilic cyanobacterium *T. elongatus*. The photobioreactor allows for control of temperature (56 °C), CO_2 and air intake (flow rates of air 1000 L/min with 40 mL/min CO_2 enrichment flow, pH control (adjusted to pH=7.0), light illumination using red and white light-emitting diodes with light gradients that automatically adjust the light intensity to the cell density. **b** Representative chromatogram of the proteins solubilized from *T. elongatus* membranes by detergent extraction. PSI trimers were separated from phycobillisomes in the flow through and PSII (monomer and dimer, which also contain traces of PSI monomer) by anion exchange chromatography (DEAE, Toyopearl) using a step gradient of $MgSO_4$ during FPLC chromatography. The elution of peaks was detected by a 3-wavelength detector monitoring the protein concentration at 280 nm (red line) and two wavelengths of light that correspond to the maximum absorption of chlorophylls in PSII at 672 nm and in PSI at 681 nm (blue and green lines, respectively).

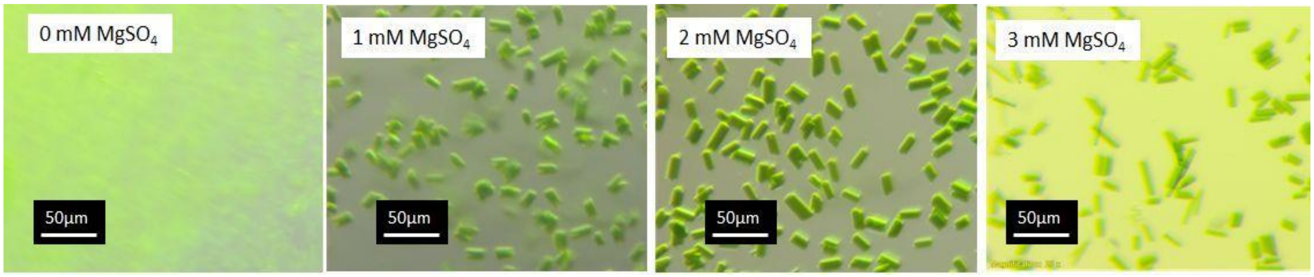

Supplementary Fig. 2 Representative results of PSI crystallization test trials in varying concentrations of $MgSO_4$ in buffer containing 5 mM MES pH=6.4 and 0.02% β-DDM. Please note that in this case only amorphous precipitate is observed in buffer without $MgSO_4$. At 1 mM $MgSO_4$ crystals of uniform size are observed but they show growth defects (like hollow crystals and crystals with rugged edges). The best crystals in this test are grown in the presence of 2 mM $MgSO_4$. At higher $MgSO_4$ concentration (3 mM) crystals become larger and grow into more needle-shaped crystals with a less uniform size distribution. Also, the crystal yield decreases as not all PSI is crystallized under these conditions (as evidenced from the green solution surrounding the crystals).

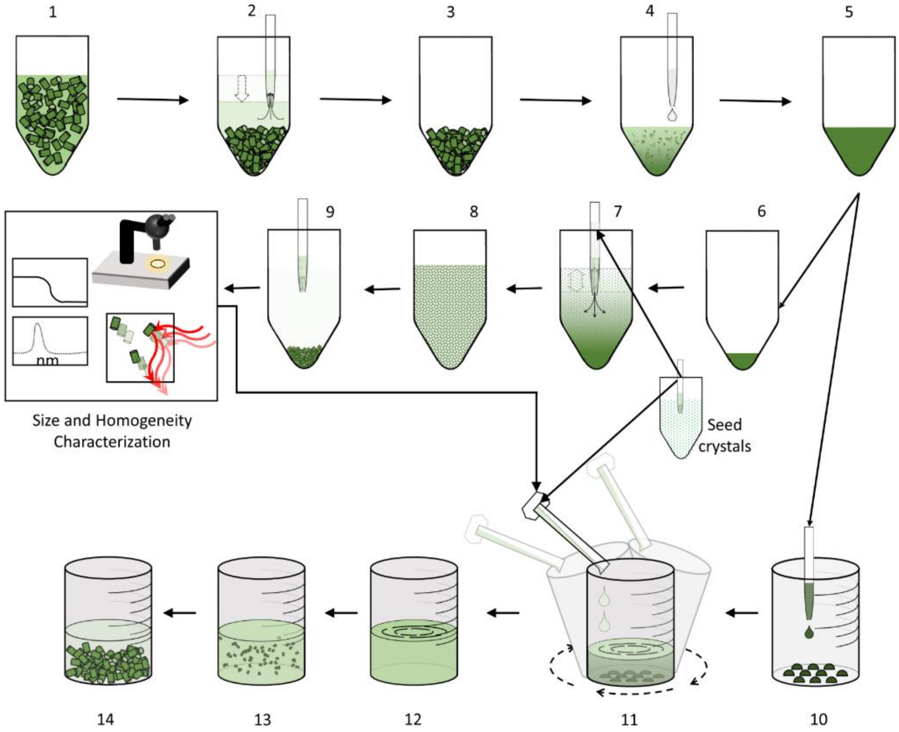

Supplementary Fig. 3 Bulk crystallization with seeding via RAMS (Rotational Agitated Mixing with Seeds).

TOP ROW: The description starts at the top left with (1) crystals of PSI of broad size distribution that had been generated by concentration of PSI at low ionic strength by ultrafiltration. (2) The crystals are pelleted by centrifugation, (3) the supernatant is removed and (4) the crystals are dissolved by addition of buffer containing 100 mM $MgSO_4$ to generate (5) a highly concentrated PSI solution at 100 mg/mL.

MIDDLE ROW: In the next line the fast test experiment is shown to determine the ideal salt concentration for crystal growth. (6) 1 µL of the PSI solution is (7) mixed with 50 µL of a precipitant solution that contain nanocrystal seeds leading to (8) a final protein concentration of 17.4 mg/mL. (9) Crystals grow in 1 hour and are monitored for quality using light microscopy.

BOTTOM ROW: The last row shows the large-scale bulk crystallization for sample delivery to the XFEL. The sequence of events is shown from the right to the left: (10) 150 µL of the PSI solution is applied to the bottom of a Nalgene beaker in the form of thirty 5 µL drops. (11) Precipitant solution containing nanoseeds of uniform size distribution is added to the beaker with gently mixing by rotation of the beaker. (12) At the final protein concentration of 17.4 mg/mL PSI, crystals of uniform size distribution (13) grow during 1-hour incubation at room temperature in the dark and (14) sediment to the bottom of the beaker from which they are harvested and used for sample delivery.

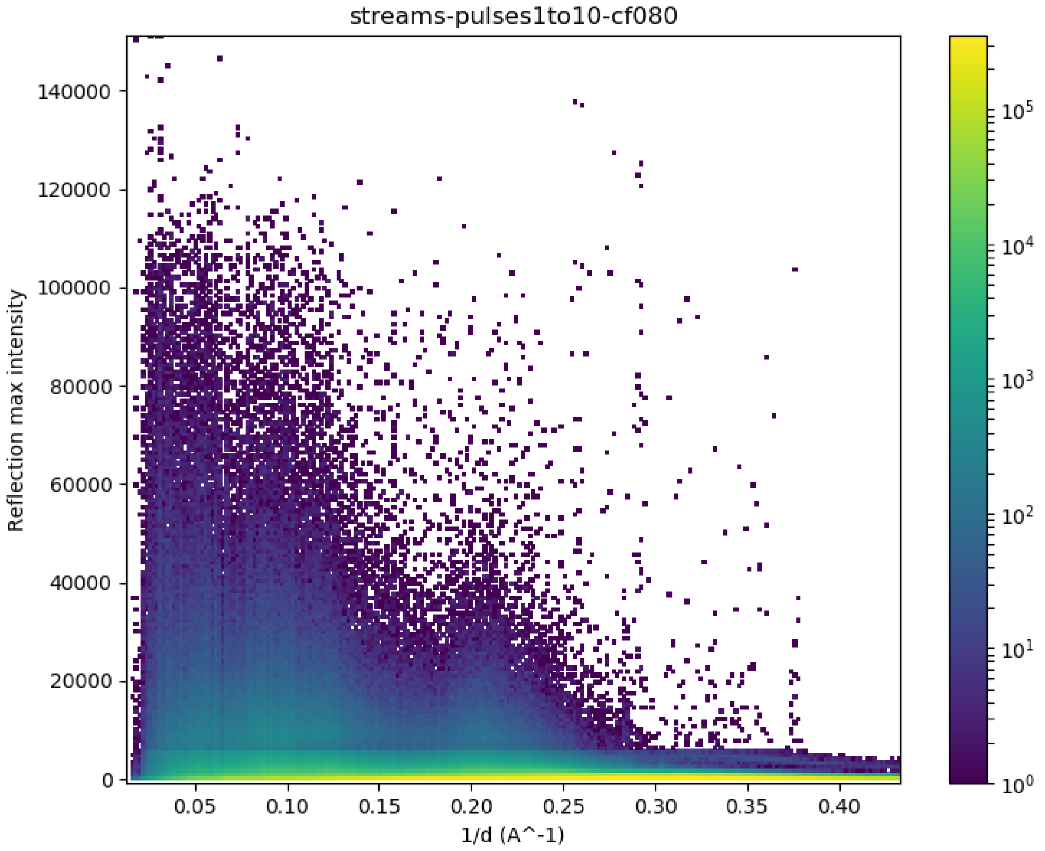

Supplementary Fig. 4 Histogram of maximum pixel intensities in indexed peaks, showing the wide dynamic range of the AGIPD. This plot was generated by *peakogram-stream*, a script included with CrystFEL[9].

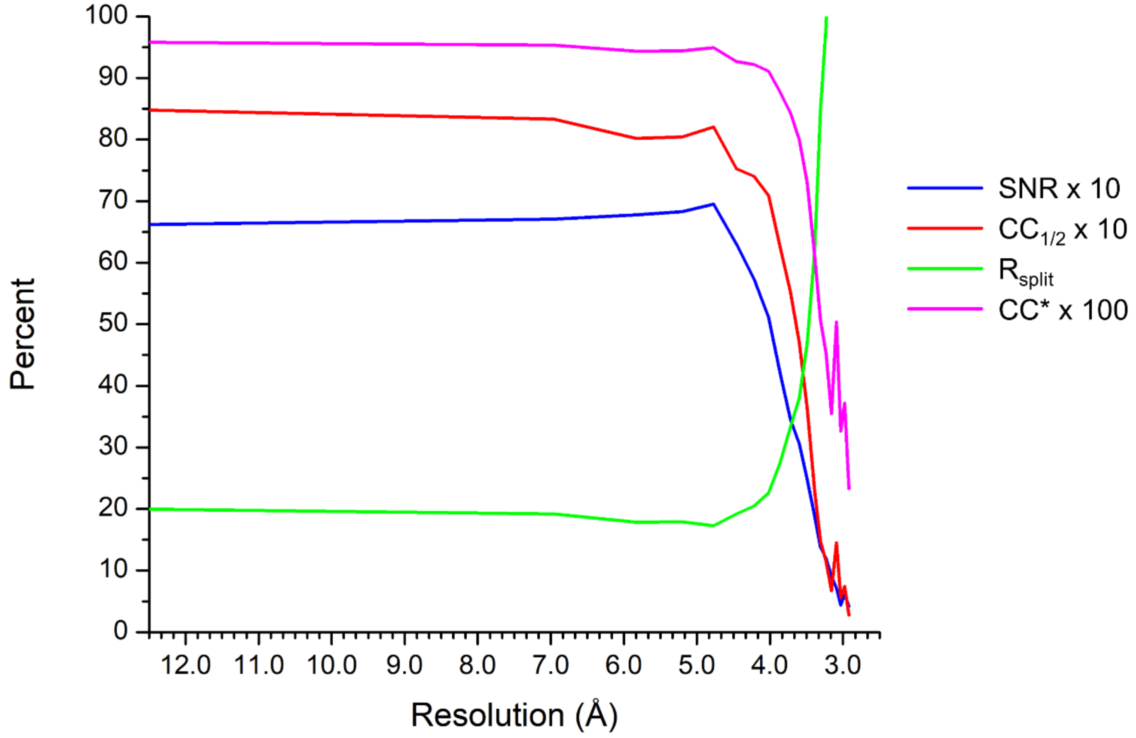

Supplementary Fig. 5 Data analysis statistics plotted as a function of resolution. R_{split} is defined in White et al. (2016)[10].

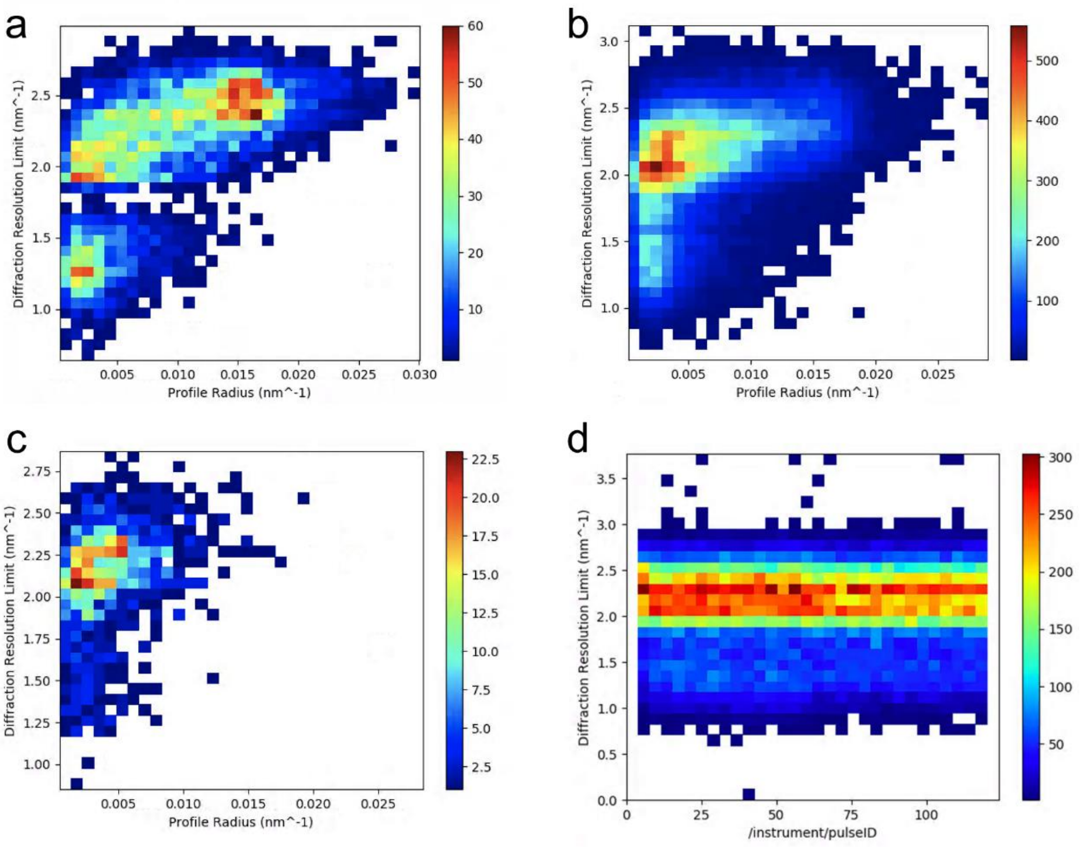

Supplementary Fig. 6 Diffraction resolution limits of SFX PSI data collected at 3 sample-to-detector distances (for all pulses) and for each pulse ID (for all distances). **a** – **c** 2D histograms of per-pattern diffraction resolution vs Bragg reflection profile radii, both of which are calculated by CrystFEL[9] automatically per pattern, with the sample-to-detector distance of **a** 0.326 m, **b** 0.233 m and **c** 0.168 m. The reflection profile radius is the minimum size of a spherical reflection radius that accounts for the found peaks in the pattern. Diffraction profile radii increase due to indexing inaccuracy, mosaicity, or bandwidth. **d** 2D histogram of the diffraction resolution for all indexed patterns for each pulse ID within the pulse train, showing that the resolution distribution does not change significantly during the pulse train. The dark structure of PSI was determined from pulses ID's 4 through 40 (10 pulses). These plots were generated with the program DatView which is available at https://github.com/nstander/DatView.

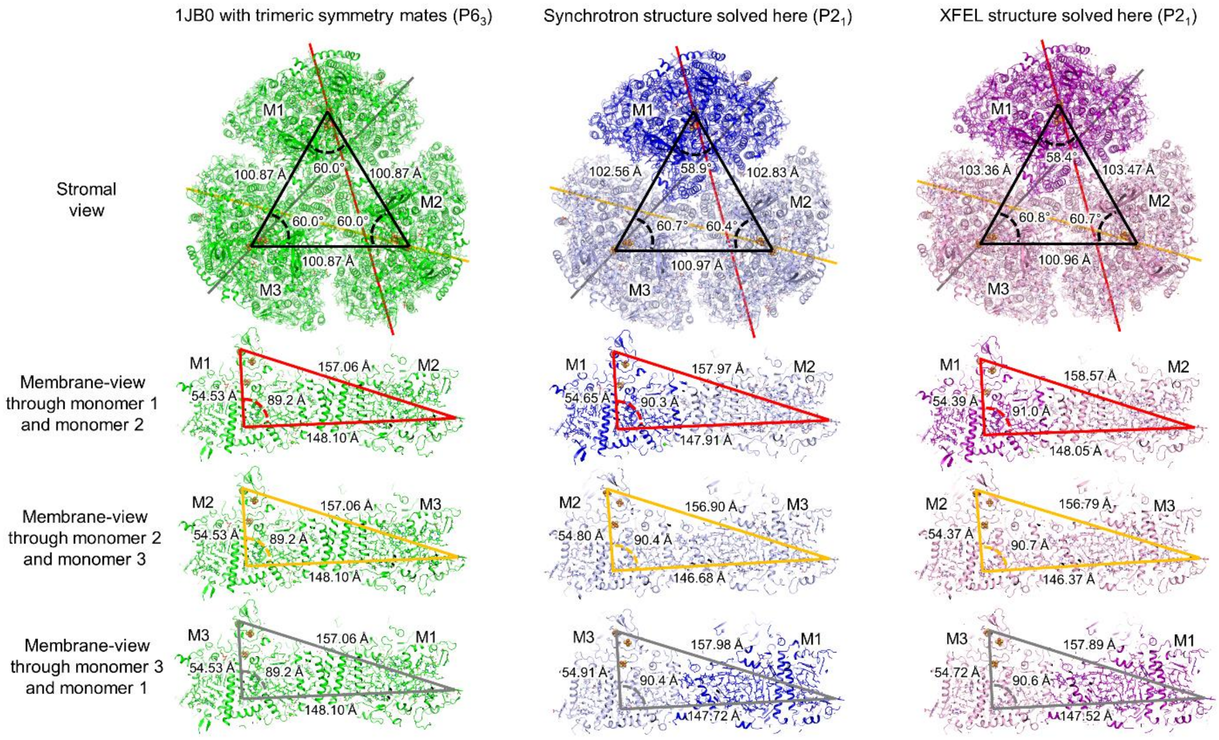

Supplementary Fig. 7 Monomer orientations within the trimeric *T. elongatus* PSI complex. A stromal view and three membrane-plane slices are shown for each of three structures. Left: The PSI structure previously solved in the P6$_3$ space group showing the symmetry mates that comprise the trimer. Middle: The cryogenic synchrotron structure solved herein. Right: The RT XFEL structure solved herein. In the stromal view (top), measurements were taken from the most peripheral atom within a 4Fe-4S cluster. In the stromal views, the red, orange, and grey lines correspond to the three slices below each. In each of the membrane-plane slices, the same atom (most peripheral atom in the 4Fe-4S clusters), the central Mg atom of the Chl *a*', and the central Mg of a peripheral Chl *a* (PsaB Chl 1233), are used to show the angle and distances. In the structures solved in P2$_1$ without symmetry, one of the monomers of the trimer is further from the other two monomers. This monomer is colored darker than the other two in the middle and right columns.

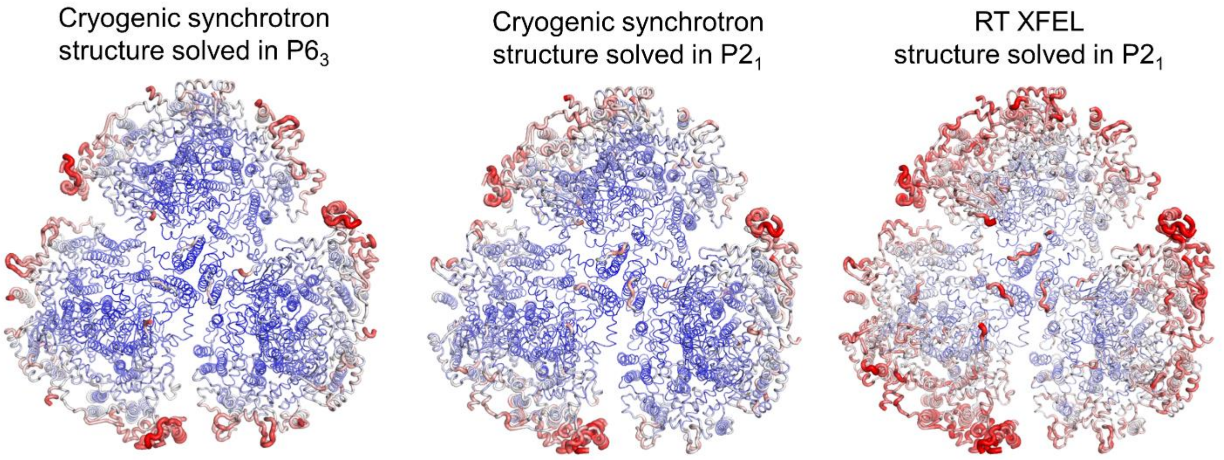

Supplementary Fig. 8 B-factor comparison of the three *T. elongatus* structures discussed in the main text. The "putty" view of the polypeptides is shown where larger and red means a higher B-factor and smaller and blue means a lower B-factor. B-factor scale was from 20 – 100 Å^2.

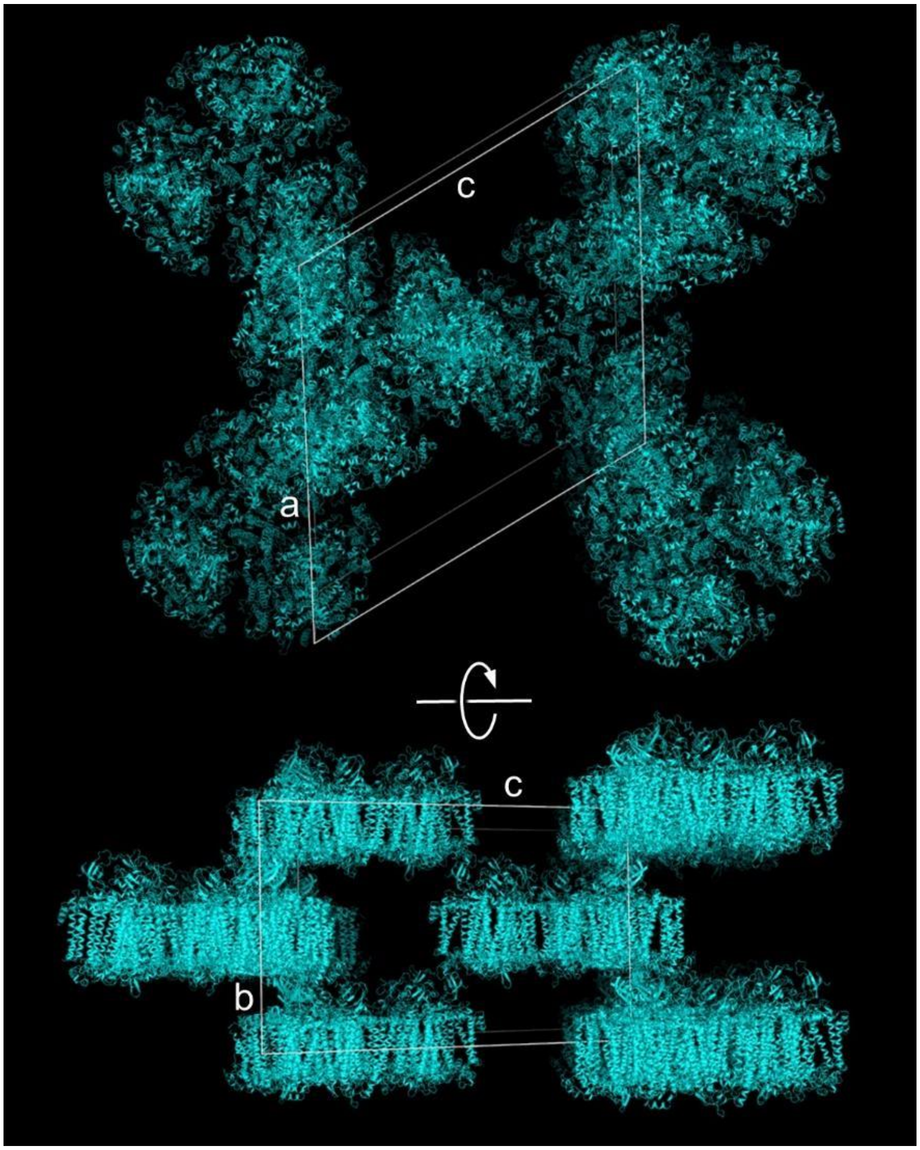

Supplementary Fig. 9 Packing of PSI in space group $P2_1$. Unit cell edges *a*, *b*, and *c* are labelled. The arrangement of PSI trimers in the $P2_1$ unit cell is shown in a view onto the a/c plane (top) and in parallel to the a/c plane (bottom). PyMOL[8] was used to generate symmetry mates within the unit cell. The membrane-normal (top) and membrane parallel (bottom) views of the protein are shown within the unit cell (white lines). Only protein secondary structure is shown for clarity.

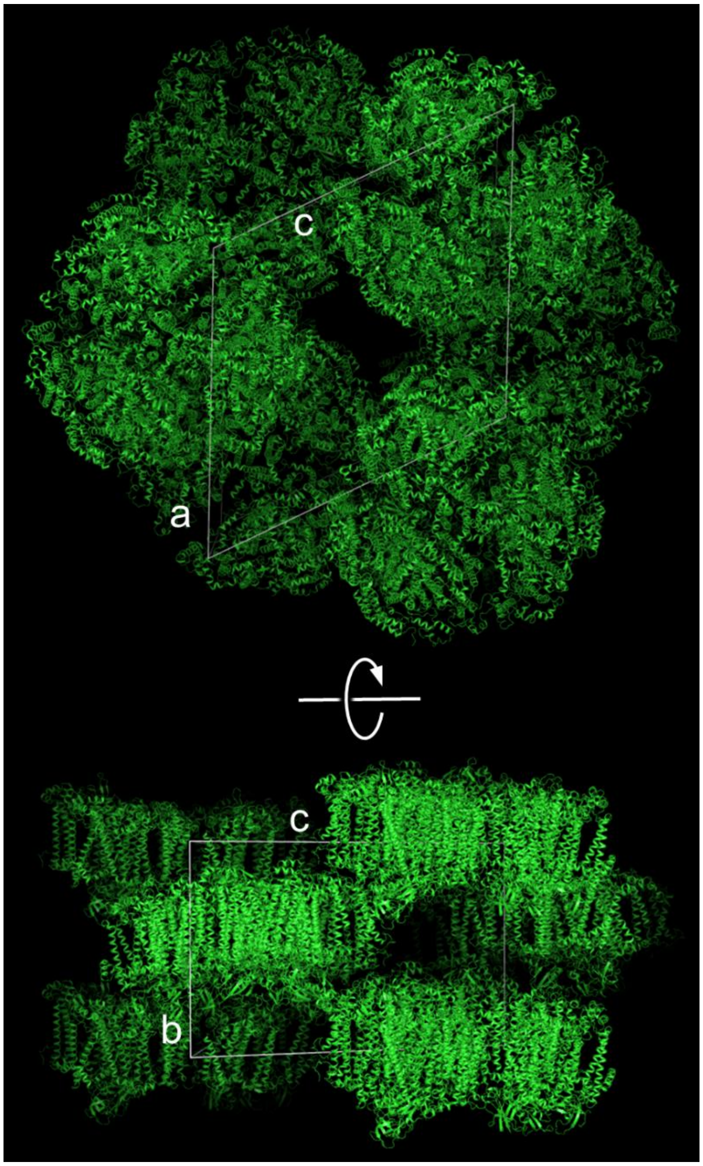

Supplementary Fig. 10 Packing of PSI in space group $P2_1$ from the structure of PSI from *S*. sp. PCC 6803 reported previously (PDB ID=5OY0)[11]. Unit cell edges *a*, *b*, and *c* are labelled. The arrangement of PSI trimers in the $P2_1$ unit cell is shown in a view onto the a/c plane (top) and in parallel to the a/c plane (bottom). PyMOL was used to generate symmetry mates within the unit cell. The membrane-normal (top) and membrane parallel (bottom) views of the protein are shown within the unit cell (while lines). Only protein secondary structure is shown for clarity.

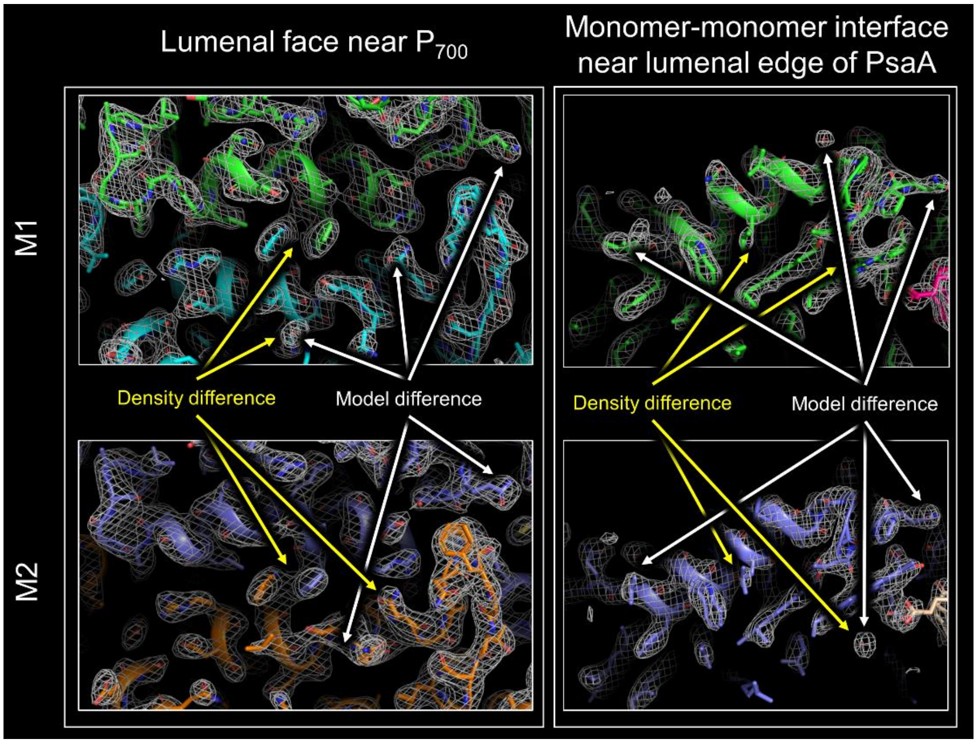

Supplementary Fig. 11 Example differences between individual monomers of the trimer without symmetry-imposed refinement of the XFEL model. The two images on the top are from “M1”, the monomer of the trimer where the core polypeptides are composed of chains A and B in the associated PDB file. The two images on the bottom are from “M2”, the monomer of the of the trimer where the core polypeptides are composed of chains G and H in the associated PDB file. On the left is a lumenal view of two TMH (one from each core polypeptide) that sits below the primary electron donor, a pair a Chl molecules called P_{700}. On the right shows a region toward the lumenal edge of PsaA near the monomer-monomer interface. Both differences in density and structure are labelled; however, note that at 2.9 Å resolution, these differences are considered negligible. The 2Fo-Fc map is shown at 1.5σ for all panels.

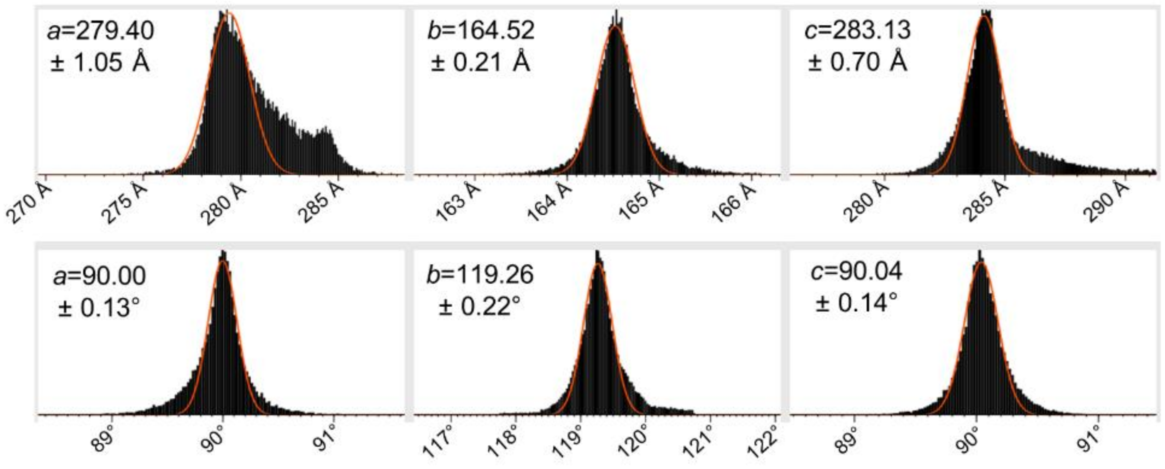

Supplementary Fig. 12 PSI microcrystal unit cell distribution. PSI microcrystals were determined by indexing EuXFEL SFX data with MOSFLM[12]. The red lines show a Gaussian function fit to the unit cell constant distribution and the corresponding peak value is listed in each sub-panel.

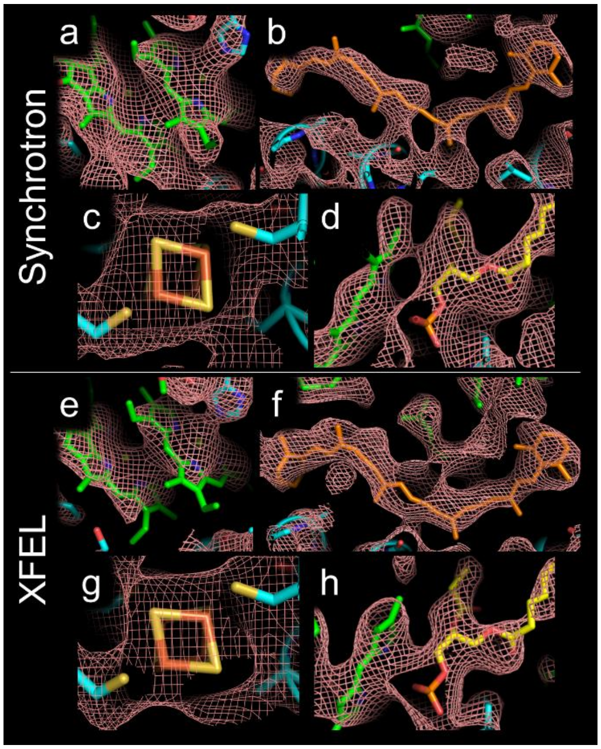

Supplementary Fig. 13 Annealed composite omit map (1σ) of the diffraction data generated in the Phenix software suite[13]. 0.5% of the atoms within the asymmetric unit were iteratively omitted and all other options were left to their default settings. In all images, protein is colored cyan, Chl molecules are colored green, β-carotenes are colored orange, lipids are colored yellow. Nitrogen atoms are additionally colored blue, oxygen atoms are additionally colored red, and magnesium atoms are additionally colored bright green. **a** and **e** show the omit map of the “special pair” of chlorophylls, “P_{700}”. **b** and **f** show the omit map a β-carotene molecule, **c** and **g** show the omit map of the 4Fe-4S cluster, “F_X”, and **d** and **h** show the omit map for a phosphatidylglycerol headgroup axial coordination of a Chl molecule.

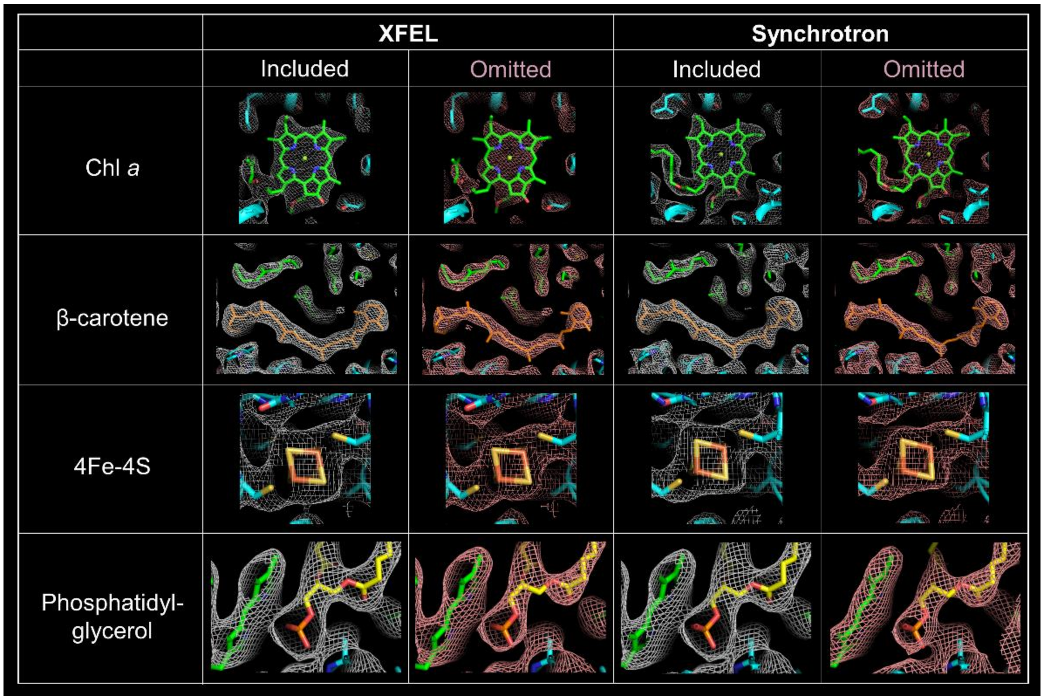

Supplementary Fig. 14 Manual omission of selected ligands from the Fo-Fc electron density map at 1σ of the XFEL- and synchrotron-derived data. In each row, the electron density map is shown after refinement with (left column, white map) and without (right column, pink map) the ligand of interest. For the map where the ligand was omitted, the full model was placed back into the electron density for visual reference. The first row shows the Chl that is the initial electron acceptor in the electron transfer chain, "A_0". The second row shows a β-carotene. The third row shows a 4Fe-4S cluster, F_X. The fourth row shows a phosphatidyl glycerol molecule whose headgroup provides the axial ligand to the central magnesium of a Chl *a*.

Bibliography

1. Jordan, P. *et al.* Three-dimensional structure of cyanobacterial Photosystem I at 2.5 Å resolution. *Nature* **411**, 909–917 (2001).

2. Fromme, P. & Witt, H. T. Improved isolation and crystallization of photosystem I for structural analysis. *Biochim. Biophys. Acta - Bioenerg.* **1365**, 175–184 (1998).

3. Kabsch, W. XDS. *Acta Crystallogr. Sect. D* **66**, 125–132 (2010).

4. Winn, M. D. *et al.* Overview of the CCP4 suite and current developments. *Acta Crystallogr. Sect. D Biol. Crystallogr.* **67**, 235–242 (2011).

5. McPherson, A. Preparation and Analysis of Protein Crystals. in *Book Reviews* (1982). doi:10.4314/ajtcam.v11i2.29

6. Allahgholi, A. *et al.* The adaptive gain integrating pixel detector at the European XFEL. *J. Synchrotron Radiat.* **26**, 74–82 (2019).

7. Henrich, B. *et al.* The adaptive gain integrating pixel detector AGIPD a detector for the European XFEL. *Nucl. Instruments Methods Phys. Res. Sect. A Accel. Spectrometers, Detect. Assoc. Equip.* **633**, S11–S14 (2011).

8. DeLano, W. L. The PyMOL Molecular Graphics System, Version 1.8. *Schrödinger LLC* http://www.pymol.org (2014). doi:10.1038/hr.2014.17

9. White, T. A. *et al.* CrystFEL: A software suite for snapshot serial crystallography. *J. Appl. Crystallogr.* **45**, 335–341 (2012).

10. White, T. A. *et al.* Recent developments in CrystFEL. *J. Appl. Crystallogr.* **49**, 680–689 (2016).

11. Malavath, T., Caspy, I., Netzer-El, S. Y., Klaiman, D. & Nelson, N. Structure and function of wild-type and subunit-depleted photosystem I in *Synechocystis*. *Biochim. Biophys. Acta - Bioenerg.* **1859**, 645–654 (2018).

12. Battye, T. G. G., Kontogiannis, L., Johnson, O., Powell, H. R. & Leslie, A. G. W. IMOSFLM: a new graphical interface for diffraction-image processing with MOSFLM. *Acta Crystallogr. Sect. D* **67**, 271–281 (2011).

13. Adams, P. D. *et al.* PHENIX: A comprehensive python-based system for macromolecular structure solution. *Acta Crystallogr. Sect. D Biol. Crystallogr.* **66**, 213–221 (2010).
