# Supplementary material for: Membrane protein megahertz crystallography at the European XFEL
Source: Nat Commun. 2019 Nov 4;10:5021. doi: 10.1038/s41467-019-12955-3 (PMC6828683; doi:10.1038/s41467-019-12955-3)
Supplement: Supplementary file 2 — Reporting Summary [file 41467_2019_12955_MOESM2_ESM.pdf]

nature research

Corresponding author(s): Petra Fromme at Petra.Fromme@asu.edu
Nadia Zatsepin at Nadia.Zatsepin@asu.edu

Last updated by author(s): Jun 26, 2019

Reporting Summary

Nature Research wishes to improve the reproducibility of the work that we publish. This form provides structure for consistency and transparency in reporting. For further information on Nature Research policies, see Authors & Referees and the Editorial Policy Checklist.

Statistics

For all statistical analyses, confirm that the following items are present in the figure legend, table legend, main text, or Methods section.

n/a	Confirmed	
☐	☒	The exact sample size (*n*) for each experimental group/condition, given as a discrete number and unit of measurement
☐	☒	A statement on whether measurements were taken from distinct samples or whether the same sample was measured repeatedly
☒	☐	The statistical test(s) used AND whether they are one- or two-sided<br>*Only common tests should be described solely by name; describe more complex techniques in the Methods section.*
☒	☐	A description of all covariates tested
☐	☒	A description of any assumptions or corrections, such as tests of normality and adjustment for multiple comparisons
☐	☒	A full description of the statistical parameters including central tendency (e.g. means) or other basic estimates (e.g. regression coefficient) AND variation (e.g. standard deviation) or associated estimates of uncertainty (e.g. confidence intervals)
☒	☐	For null hypothesis testing, the test statistic (e.g. *F*, *t*, *r*) with confidence intervals, effect sizes, degrees of freedom and *P* value noted<br>*Give P values as exact values whenever suitable.*
☒	☐	For Bayesian analysis, information on the choice of priors and Markov chain Monte Carlo settings
☒	☐	For hierarchical and complex designs, identification of the appropriate level for tests and full reporting of outcomes
☒	☐	Estimates of effect sizes (e.g. Cohen's *d*, Pearson's *r*), indicating how they were calculated

Our web collection on statistics for biologists contains articles on many of the points above.

Software and code

Policy information about availability of computer code

Data collection	N/A
Data analysis	The only unpublished code used in this study was that from Xgandalf to determine the unit cell parameters of the XFEL data. This code is currently available at https://stash.desy.de/users/gevorkov/repos/xgandalf and will be included in the CrystFEL software suite (http://www.desy.de/~twhite/crystfel/) upon its next update.

For manuscripts utilizing custom algorithms or software that are central to the research but not yet described in published literature, software must be made available to editors/reviewers. We strongly encourage code deposition in a community repository (e.g. GitHub). See the Nature Research guidelines for submitting code & software for further information.

Data

Policy information about availability of data

All manuscripts must include a data availability statement. This statement should provide the following information, where applicable:
- Accession codes, unique identifiers, or web links for publicly available datasets
- A list of figures that have associated raw data
- A description of any restrictions on data availability

The PDB accession code for the XFEL structure is 6PGK. The PDB accession code for the synchrotron structure is 6PFY.
Figures 1, 2, 3, S1, S5, S6, S8, S9, S13, and S14 have associated raw data.
There are no restrictions on data availability. XFEL-collected crystallography data will be deposited into the Coherent X-ray Imaging Data Bank upon acceptance.
Synchrotron-collected crystallography data will be available in the Protein Data Bank upon acceptance.

Field-specific reporting

Please select the one below that is the best fit for your research. If you are not sure, read the appropriate sections before making your selection.

☒ Life sciences ☐ Behavioural & social sciences ☐ Ecological, evolutionary & environmental sciences

For a reference copy of the document with all sections, see nature.com/documents/nr-reporting-summary-flat.pdf

Life sciences study design

All studies must disclose on these points even when the disclosure is negative.

Sample size	Data were collected from 1000 mg of crystals leading to 59,012 indexed diffraction patterns.
Data exclusions	All diffraction patterns that were collected were included in the analysis.
Replication	To verify the unit cell parameters for the XFEL data derived from the Xgandalf program, the same parameters were determined using the MOSFLM program. Furthermore, the unit cell was confirmed by solving the cryogenic synchrotron structure.
Randomization	Only non-excited data was included in the analysis as described in the main text.
Blinding	The unit cell parameters were unknown prior to the experimentation.

Reporting for specific materials, systems and methods

We require information from authors about some types of materials, experimental systems and methods used in many studies. Here, indicate whether each material, system or method listed is relevant to your study. If you are not sure if a list item applies to your research, read the appropriate section before selecting a response.

Materials & experimental systems

n/a	Involved in the study	
☒	☐	Antibodies
☒	☐	Eukaryotic cell lines
☒	☐	Palaeontology
☒	☐	Animals and other organisms
☒	☐	Human research participants
☒	☐	Clinical data

Methods

n/a	Involved in the study	
☒	☐	ChIP-seq
☒	☐	Flow cytometry
☒	☐	MRI-based neuroimaging
